# Docosahexaenoic acid remodels lipid composition of lipid rafts and mitochondrial function in ApoE4/4 mouse brain: a multi-omic investigation

**DOI:** 10.1186/s43556-026-00618-x

**Published:** 2026-10-15

**Authors:** Yu Liu, Jingjing Xu, Xixiang Wang, Lu Liu, Xiuwen Ren, Yunyun Gong, Chen Miao, Mengyang Sun, Shaobo Zhou, Xinjing Guo, Linhong Yuan

**Affiliations:** 1 School of Public Health, Capital Medical University; Beijing Key Laboratory of Environment and Aging; China-British Joint Laboratory of Nutrition Prevention and Control of Chronic Diseases, You Anmen Street, Beijing, 100069 China; 2 School of Food Science and Nutrition, University of Leeds, Woodhouse Lane, West Yorkshire, Leeds, LS2 9JT UK; 3 School of Science, Faculty of Engineering and Science, University of Greenwich, Central Avenue, Chatham, ME4 4TB UK

**Keywords:** Alzheimer’s disease, Apolipoprotein E, Docosahexaenoic acid, Lipid raft, Mitochondrion

## Abstract

Alzheimer’s disease (AD) is an irreversible neurodegenerative disorder lacking curative therapies, with ApoE4 as the strongest genetic risk factor driving lipid dyshomeostasis and neuronal damage in the central nervous system. Lipid rafts, cholesterol- and sphingolipid-rich membrane microdomains, govern key neuronal signalling, and dysfunction of these microdomains mediates the progression of preclinical pathological lesions in ApoE4 carriers. Dietary docosahexaenoic acid (DHA) is widely reported to mitigate brain lipid disorders, yet how DHA remodels subfractions of lipid raft and non-raft membrane domains to rescue ApoE4-related energy metabolism disorder remains unclear. Most existing studies have only quantified total brain lipids without distinguishing membrane subdomains, and few characterized the liver-brain metabolic axis underlying ApoE4-associated neurodegeneration. We constructed female ApoE3/3 and ApoE4/4 knock-in mice with 6- and 12-month DHA dietary interventions, combining behavioral, histological, lipidomic and proteomic assays to characterize genotype-specific lipid remodelling and downstream mitochondrial changes. Here we show that ApoE4 mice develop severe hepatic steatosis, neuroinflammation and lipid raft rigidification characterized by elevated sulfatides and ceramides levels, DHA upregulates *Lpin1*, *Atgl* and *nSMase2* while suppressing *Acer3* and *Sgms2*, thereby rebalancing lipids raft and restoring mitochondrial bioenergetics. Our work resolves raft and non-raft lipid profiles in ApoE humanized mice, and links peripheral hepatic metabolic disturbances to cerebral membrane microdomain dysfunction. These multi-omics mechanistic findings advance genotype-specific preventive nutritional strategies for ApoE4 carriers and reveal potential subcellular lipid targets for early AD intervention.

**Supplementary Information:**

The online version contains supplementary material available at https://doi.org/10.1186/s43556-026-00618-x.

## Introduction

Alzheimer’s disease (AD), a neurodegenerative disorder with irreversible memory and cognitive decline [1]. AD accounts for 60%−80% of dementia cases, affecting 5% of individuals aged 65 and above [2, 3]. Etiology of AD is complex, with hypotheses including β-amyloid (Aβ) cascade, tau protein hyperphosphorylation, disrupted cholesterol homeostasis, mitochondrial dysfunction, and insulin resistance [2]. Currently, there are no effective treatments for AD, intervening in modifiable risk factors is a promising strategy to reduce AD incidence.

Apolipoprotein E (ApoE), the primary lipid carrier in the central nervous system, is predominantly secreted by astrocytes and microglia. It transports cholesterol and other lipids to neurons by binding to cell surface ApoE receptors. ApoE *ε*4 represents the strongest genetic risk factor for AD [4]: carriers of one *ε*4 allele exhibit a fourfold increased AD, while ApoE *ε*4/*ε*4 carriers have a 12-fold elevated risk compared to ApoE *ε*3 carriers [5]. Amino acid variations in ApoE alleles modify receptors affinity and lipid clearance [6]. ApoE genetic modify secretase activity in neuronal lipid rafts, thereby influencing Aβ production and clearance and contributing to AD pathology [7].

Docosahexaenoic acid (DHA) is a crucial n-3 polyunsaturated fatty acid (n-3 PUFA), constituting 35%−40% of the total brain PUFA. DHA promotes neurodevelopment, maintains membrane integrity and fluidity, and cerebral growth [8]. Endogenous synthesis of DHA via metabolism of α-linolenic acid is insufficient, necessitating dietary supplementation [9]. DHA is predominantly esterified with phosphatidylethanolamine (PE) and phosphatidylserine (PS) into glycerophospholipids (GP) and accumulates in neuronal membranes and synaptic vesicles [10]. Decreased neuronal membrane DHA impairs learning and memory ability [11]. Brain DHA levels of aged rats are diminished, but can be restored after one month on a fish oil diet containing 11% DHA [12]. AD patients have markedly decreased cerebral DHA, accompanied by structural alterations in lipid rafts [13].

Lipid rafts are rich in cholesterol and sphingolipids, located on cell membranes and internal membrane systems. They either float freely within the disordered plasma membrane bilayer or aggregate to form larger, more ordered platforms, comprising 1% ~5% of the total membrane area, including planar lipid rafts (2–20 nm) and flask-shaped caveolae (50–200 nm) [14]. They selectively incorporate or exclude proteins, serving as critical platforms for cellular signal transduction, ion channel function, material transport, and protein sorting [15]. Their formation may be attributed to the unsaturated state of phospholipid fatty acids in non-lipid rafts, while the hydrocarbon chains in sphingomyelins (SM) within lipid rafts achieve a saturated configuration [16]. Brain lipid raft lipids undergo age-dependent changes, accompanied by decrease in cholesterol, cholesterol esters (CE) and long-chain PUFA, but an increase in SM and saturated fatty acids along with an elevated phospholipid/cholesterol ratio. These changes diminish lipid raft fluidity [17], decrease presynaptic vesicle fusion, alter neurotransmitter release, and promote amyloid cascade-related protein interactions, facilitating Aβ aggregation and formation [18]. Consequently, studying and maintaining the lipid rafts function is crucial for the prevention of AD.

This study explores the differences in learning-memory, neuroinflammation, central Aβ metabolism and lipid metabolism in ApoE4 and ApoE3 mice following a DHA dietary intervention; clarify how ApoE4 and DHA influence membrane lipid composition of lipid raft and mitochondria function during aging. The findings will provide scientific evidence to support the development of ApoE genotype-specific nutritional strategies for dietary prevention of AD.

## Results

### Effect of DHA on body weight and learning and memory ability in ApoE3 and ApoE4 mice

**Fig. 1:**
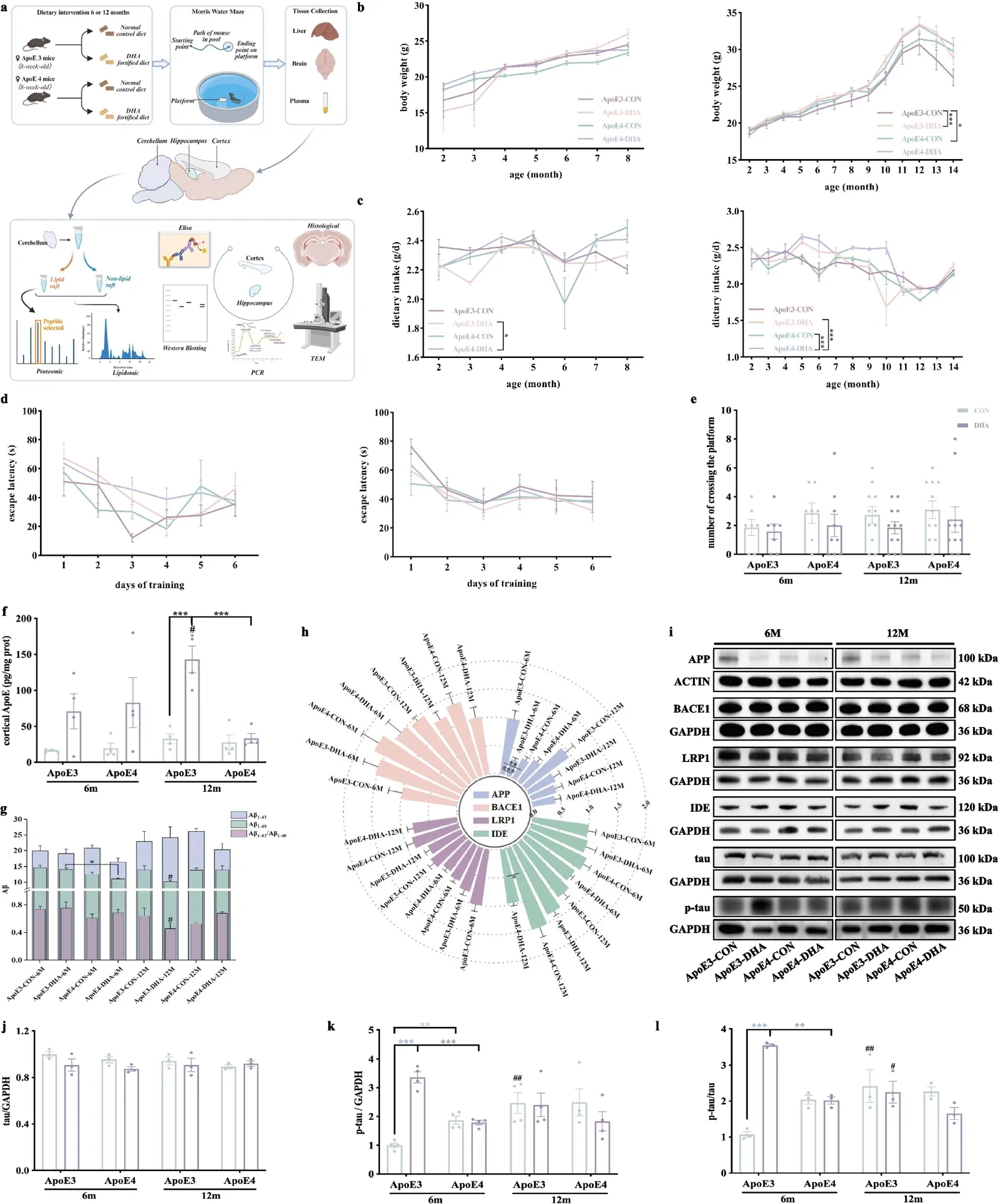
Growth, Behavioral Performance, and Cortical AD-Related Marker Levels in Mice. **a** Schematic overview of the experimental design. **b**-**c** Changes in body weight (**b**) and dietary intake (**c**) of mice in each group during the intervention period (*N* ≥ 7, mean ± SEM). **d**-**e** Morris water maze performance: acquisition phase (**d**) and probe trial (**e**) (*N* ≥ 7, mean ± SEM). **f** Cortical ApoE levels (pg/mg protein) (*N* = 4). **g**: Cortical Aβ_1–42_, Aβ_1–40_ and Aβ_1–40/Aβ1–42_ ratios (pg/mg protein) (*N* = 4). **h**–**l** Cortical expression of proteins related to Aβ synthesis and degradation (**h**), tau protein and phosphorylated tau(**j**-**l**) (*N* ≥ 3). ApoE, apolipoprotein E; Aβ, amyloid-beta protein; APP, amyloid precursor protein; BACE1, β-site APP cleaving enzyme 1; LRP1, low-density lipoprotein receptor-related protein 1; IDE, insulin-degrading enzyme; TAU, tau protein. Statistical significance: *** *P* < 0.001, ** *P* < 0.01, * *P* < 0.05, ^###^ *P* < 0.001, ^##^ *P* < 0.01, ^#^ *P* < 0.05 *vs.* corresponding group after 6-month intervention

The present study monitored body weight and food intake of mice throughout the dietary intervention to observe general growth status, and behavioral assessments were performed by using the Morris water maze to measure the cognitive alterations in mice. ApoE3 and ApoE4 mice were fed a DHA diet for 6 and 12 months, respectively (Fig. 1a). During the 6-month intervention period, body weight of mice increased with age, whereas weight loss occurred from the 12-month time point in the DHA interventional group. Across the 12-month intervention, the ApoE3-CON mice displayed a lower average body weight than mice from other groups (Fig. 1b, *P* < 0.05).

During the 6 months dietary intervention, the average daily food intake was lower in ApoE3-DHA mice than in ApoE4-DHA mice. The mice from the 12-month interventional group exhibited greater fluctuations in food intake, with mice from ApoE3-DHA and ApoE4-DHA groups showing a rapid decline at 10 and 11 months old. Throughout the intervention, the mice from ApoE4-DHA group showed a higher average daily food intake than mice from ApoE3-DHA and ApoE4-CON groups (Fig. 1c, *P* < 0.05).

As shown in Fig. 1d-e, mice in both 6- or 12-month intervention groups demonstrated a gradual progressive decrease in escape latency from day 1 to day 6. Both ApoE3 and ApoE4 mice that underwent 6 or 12 months of DHA intervention displayed a declining trend in platform-crossing frequencies.

In summary, DHA intervention induced different changes in body weight and food intake among mice with distinct ApoE genotypes, and the mice from DHA-treated groups exhibited a trend of fewer platform crossings times in the Morris water maze.

### Effect of DHA on cortical AD pathology-related markers in ApoE3 and ApoE4 mice

ELISA and Western Blotting methods were conducted to detect ApoE, Aβ, APP, tau and phosphorylated tau (p-tau) protein expressions for investigating differences in AD-related pathological markers. After dietary intervention for 12 months, cortical ApoE levels in ApoE3 mice were consistently increased, and an interaction between ApoE genotype and diet on cortical ApoE level was observed (Table S1−2). Following aging of mice, 12-month DHA intervention increased cortical ApoE level in ApoE3 mice (Fig. 1f, *P* = 0.03).

Cortical Aβ_1–40_ level in ApoE4-DHA mice was lower than that in the ApoE3-DHA group. In ApoE3-DHA mice, cortical Aβ_1–40_ level and Aβ_1–40_/Aβ_1–42_ ratio showed decreased trend in the 12-month group than 6-month group (Fig. 1g, *P* < 0.05). An interaction between ApoE genotype and diet on the cortical Aβ_1–40_/Aβ_1–42_ ratio was detected after 12 months of intervention (Table S1−2)*.*

After 6 months of intervention, cortical APP protein expression of ApoE4-CON mice was lower than that in ApoE3-CON mice, and 6-month DHA intervention down-regulated cortical APP protein expression in ApoE3 mice. An interaction between ApoE genotype and diet on cortical APP protein expression was observed at 6 months. After 12 months of intervention, cortical IDE protein expression was down-regulated in ApoE4 mice (Fig. 1h-i, *P* = 0.03), with an interaction between ApoE genotype and diet also noted (Table S1−3).

ApoE4-CON mice displayed higher cortical p-tau protein levels than ApoE3-CON mice at 8 months of age. A 6-month DHA supplemented diet increased both cortical p-tau expression and the p-tau/tau ratio in ApoE3 mice. As the mice aged, p-tau protein expression increased in ApoE3-CON mice (Fig. 1i-l, *P* < 0.05). Additionally, an interaction between ApoE genotype and diet on cortical p-tau protein expression and the p-tau/tau ratio was identified (Table S1−3).

Significant genotype-diet interactions were observed for cortical AD pathology -related markers and DHA exerted divergent regulatory effects across different interventional durations on AD pathology-related proteins in ApoE3 and ApoE4 mice.

### Genotype-specific neural and hepatic protective outcomes of long-term DHA intervention

**Fig. 2:**
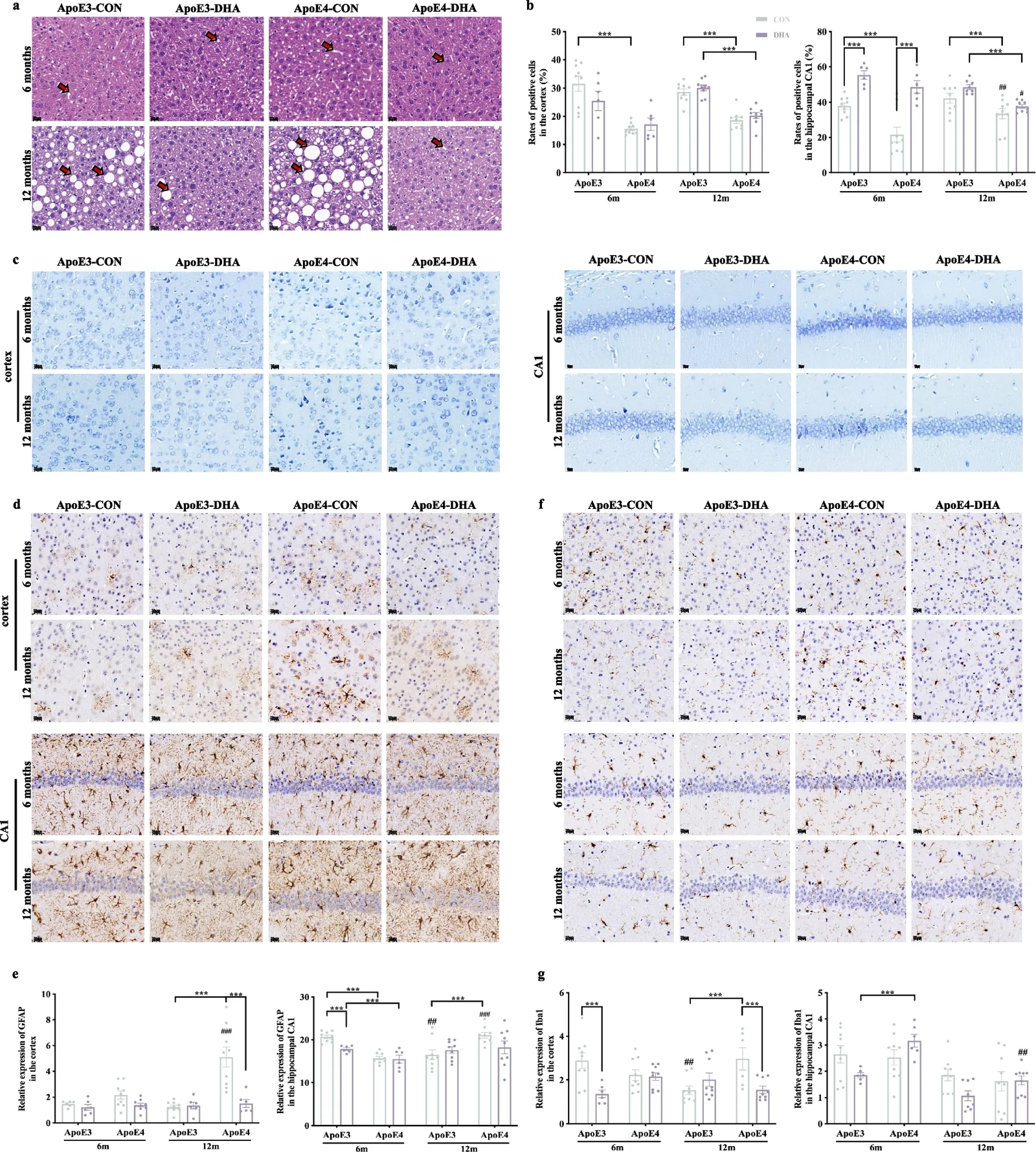
Hepatic Morphology and Neuroinflammatory Marker Expression in Mouse Brain Regions. **a** HE staining of the liver of mice in each group. **b**-**c** Nissl staining and rate of positive cells (%) in the cerebral cortex and the hippocampal CA1 region of mice. **d**-**e** GFAP protein expression in the cerebral cortex and the hippocampal CA1 region of mice. **f**-**g** Iba1 protein expression in the cerebral cortex and the hippocampal CA1 region of mice. All data represent nine repeated measurements; mean ± SEM; magnification 400 ×; scale bar: 20μm*.* GFAP, glial fibrillary acidic protein; Iba1, ionized calcium—binding adapter molecule 1. Statistical significance: *** *P* < 0.001; ^###^ *P* < 0.001, ^##^ *P* < 0.01, ^#^ *P* < 0.05 *vs.* the corresponding group with 6-month intervention

Liver HE staining, cerebral Nissl staining, and GFAP/Iba1 immunohistochemistry were applied to assess hepatic and cerebral pathological injuries in mice. As shown in Fig. 2a, after 6 months of dietary intervention, ApoE3-CON mice showed intact hepatocytes, with uniformly pink-stained liver parenchyma, distinct hepatocyte cord boundaries, and large, centrally located nuclei. ApoE4-CON mice displayed well-defined hepatocyte cords with small intracellular lipid droplets. However, ApoE4 mice treated with a DHA-fortified diet maintained intact hepatic cords and experienced a significant reduction in intracellular lipid droplets. After 12 months of intervention, ApoE3-CON mice showed marked hepatocytes swelling, indistinct cytoplasmic boundaries, and intracellular lipid droplets in most cells. ApoE4-CON mice exhibited more pronounced hepatocytes swelling, obscured cytoplasmic boundaries, disorganized hepatic cords, and numerous larger intracellular lipid droplet, presenting severe hepatic steatosis with lipid droplets predominantly distributed around hepatic sinusoids. 12 months of DHA dietary intervention reduced hepatic lipid droplets in both ApoE3 and ApoE4 mice, with a more pronounced effect in ApoE4 mice.

Compared with ApoE3 mice, ApoE4 mice showed a lower percentage of Nissl‑positive neurons in the cerebral cortex and hippocampus. In the hippocampal CA1 region, 6-month DHA intervention increased the proportion of Nissl-positive neurons relative to controls, and ApoE4-CON mice showed more positive neurons at 12 months than at 6 months of intervention (Fig. 2b-c, *P* < 0.05).

Cortical GFAP levels were markedly lower than that in hippocampus. ApoE4-CON mice exhibited higher cortical GFAP expression than ApoE3-CON mice, and the 12-month DHA intervention decreased cortical GFAP in ApoE4 mice. ApoE3-CON mice showed higher hippocampal GFAP expression than ApoE4-CON mice, and the 6-month DHA intervention reduced hippocampal GFAP expression in ApoE3 mice (Fig. 2d-e, *P* < 0.05). With aging, ApoE4-CON mice displayed higher hippocampal GFAP than ApoE3-CON mice. Genotype‑diet interaction existed for cortical GFAP at 12 months and hippocampal GFAP at 6 months (Table S1−4). Compared with the 6-month group, ApoE4-CON mice showed increased cortical GFAP after 12-month intervention (Fig. 2d-e, *P* < 0.001).

Six- and 12-month interventions down-regulated cortical Iba1 protein expression in ApoE3 and ApoE4 mice. At 12 months, ApoE4-CON mice displayed higher cortical Iba1 protein expression than ApoE3-CON mice. Within the 6-month intervention groups, hippocampal Iba1 protein expression was higher in ApoE4-DHA mice than in ApoE3-DHA mice. With aging, cortical Iba1 expression decreased in ApoE3-CON mice, while hippocampal Iba1 expression decreased in ApoE4-DHA mice (Fig. 2f-g, *P* < 0.05). Interaction effects of genotype × diet were observed for cortical Iba1 protein expression after 6- and 12-month dietary interventions, and for hippocampal after 6-month intervention (Table S1).

Taken together, DHA alleviated genotype-dependent hepatic steatosis, neuronal loss and astrocytic/microglial overactivation in a time-dependent manner, with significant genotype-diet interactions detected.

### ApoE‑genotype‑dictated lipidomic alterations of cerebellar membrane microdomains upon DHA intervention

**Fig. 3:**
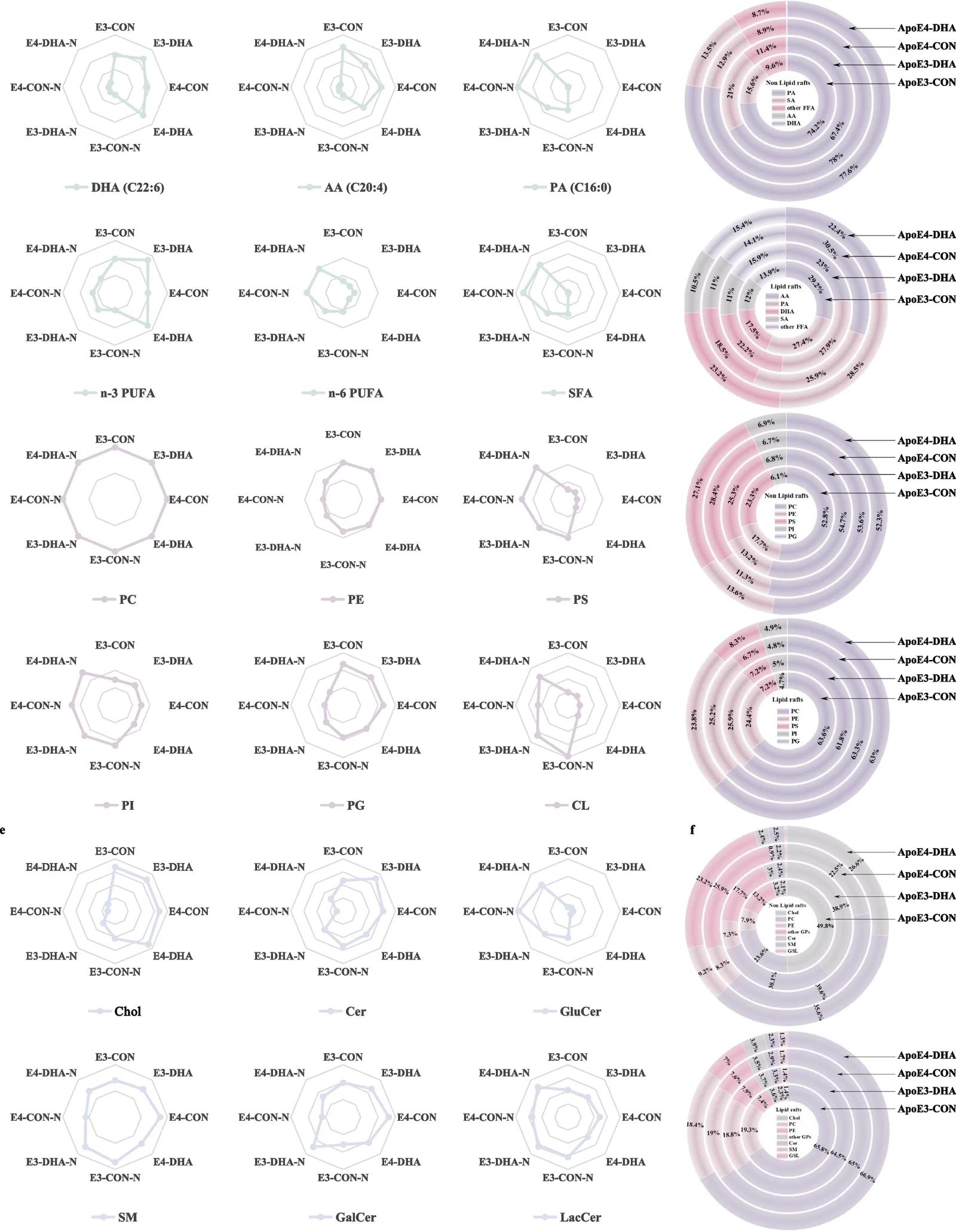
Lipid Composition of Lipid Raft and Non-Raft Domains in Mouse Brain After 12-Month Dietary Intervention. **a**-**b** Relative content (**a**) and percentage (**b**) of FFAs in brain lipid rafts and non-lipid rafts. **c**-**d** Relative content (**c**) and percentage (**d**) of GPs in brain lipid rafts and non-lipid rafts. **e**–**f** Relative content of cholesterol and sphingolipids (**e**) and the lipid composition (**f**) in brain lipid rafts and non-lipid rafts, *N* = 4. E3-CON: ApoE3 control mice, lipid raft fraction; E3-DHA: ApoE3 mice, DHA treatment, lipid raft fraction; E4-CON: ApoE4 control mice, lipid raft fraction; E4-DHA: ApoE4 mice, DHA treatment, lipid raft fraction; E3-CON-N: ApoE3 control mice, non-lipid raft fraction; E3-DHA-N: ApoE3 mice, DHA treatment, non-lipid raft fraction; E4-CON-N: ApoE4 control mice, non-lipid raft fraction; E4-DHA-N: ApoE4 mice, DHA treatment, non-lipid raft fraction. PA, phosphatidic acid; SA, stearic acid; FFA, free fatty acid; AA, arachidonic acid; DHA, docosahexaenoic acid; PUFA, polyunsaturated fatty acid; PC, phosphatidylcholine; PE, phosphatidylethanolamine; PS, phosphatidylserine; PI, phosphatidylinositol; PG, phosphatidylglycerol; Chol, cholesterol; GP, glycophospholipid; Cer, ceramide; SM, sphingomyelin; GSL, glycosphingolipid

After isolating lipid raft and non-lipid raft fractions, lipidomic profiling was performed to compare lipid signatures across all experimental groups (Fig. 3, Table S2). Lipid microdomains exhibited intrinsic lipid specificity: n‑3 PUFAs, DHA and arachidonic acid (AA) were predominantly enriched in lipid rafts, whereas saturated fatty acids (SFA) and n‑6 PUFAs preferentially accumulated in non‑lipid raft fractions.

Baseline lipid homeostasis exhibited stable genotypic differences between ApoE3 and ApoE4 mice. In non-lipid raft fractions, ApoE4 mice had higher levels of palmitic acid (PA), SFA and n-6 PUFA content relative to ApoE3 mice (*P* < 0.05). The two genotypes also differed intrinsically in basal free fatty acid (FFA) composition, GPs distribution, cholesterol abundance, and sphingolipid profiles across cerebellar lipid raft and non-raft compartments, establishing distinct baseline membrane lipid signatures.

DHA intervention induced compartment- and genotype-specific alterations in fatty acid metabolism. In lipid raft fractions, DHA supplementation effectively enriched n-3 PUFA accumulation and decreased AA content in both ApoE3 and ApoE4 mice, with ApoE4 mice showing more robust lipid responses (*P* < 0.05). In non-lipid raft fractions, DHA exerted opposing regulatory effects: it elevated SFA and n-6 PUFA levels in ApoE3 mice, whereas it reduced PA in ApoE4 mice (Fig. 3a-b).

Consistent genotype-dependent remodeling was also observed in GPs, cholesterol and sphingolipids. In non-lipid rafts, DHA reduced cardiolipin (CL) and PE levels and increased PS content in ApoE3 mice, while increasing CL and PE but decreasing phosphatidylcholine (PC) and PS in ApoE4 mice. DHA exerted divergent regulation on sphingolipids: it increased multiple ceramide subtypes in ApoE3 mice but significantly suppressed GluCer abundance in ApoE4 non-lipid rafts (*P* < 0.05) (Fig. 3c-f).

Collectively, DHA induced distinct, genotype-dependent remodeling of fatty acids, GPs, and sphingolipids within cerebellar lipid-raft and non-lipid-raft compartments of ApoE3 and ApoE4 mice.

### Compositional differences of discriminating lipids in cerebellar lipid microdomains under DHA intervention

**Fig. 4:**
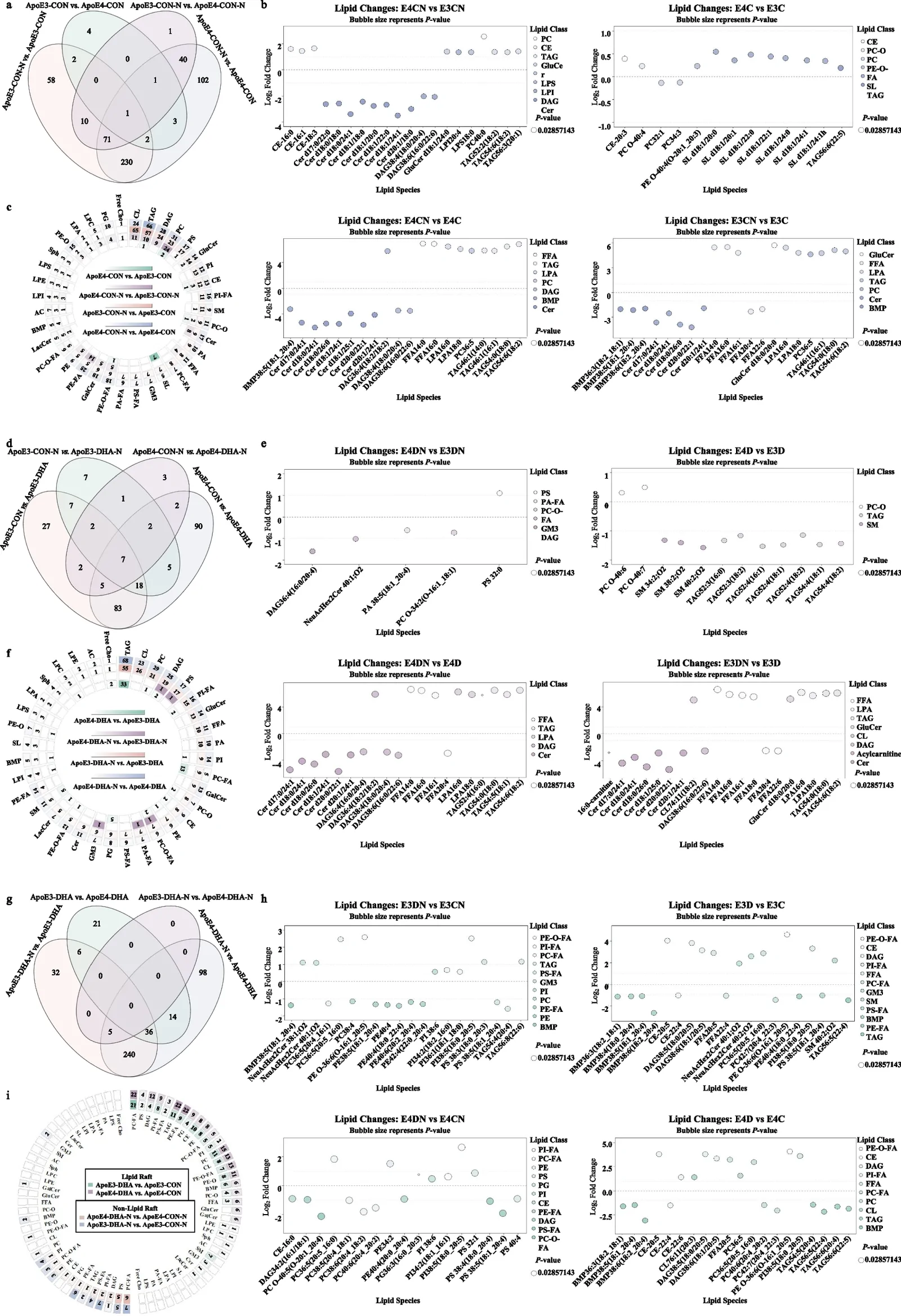
Lipidomic Profiling of Differentially Expressed Lipids in Mouse Brain Tissue. **a**-**c** After 12 months of a normal control dietary, differentially expressed lipids identified by lipidomic analysis (**a**), top 10 downregulated and top 10 upregulated lipids of mouse brain tissues (**b**) and the number of lipid categories of intergroup differential substances (**c**). **d**-**f** After 12 months of a DHA-supplemented diet, differentially expressed lipids identified by lipidomic (**d**), top 10 downregulated and top 10 upregulated lipids of mouse brain tissues (**e**), and number of lipid categories of intergroup differential substances (**f**). **g** Differentially expressed lipids identified by lipidomic between the two dietary interventions. **h** Top 10 downregulated and top 10 upregulated lipids of mouse brain tissues between the two dietary interventions. **I** the number of lipid categories among intergroup differential substances between the two dietary interventions, *N* = 4. E3C, E3-CON: ApoE3 control mice, lipid raft fraction; E3D, E3-DHA: ApoE3 mice, DHA treatment, lipid raft fraction; E4C, E4-CON: ApoE4 control mice, lipid raft fraction; E4D, E4-DHA: ApoE4 mice, DHA treatment, lipid raft fraction; E3CN, E3-CON-N: ApoE3 control mice, non-lipid raft fraction; E3DN, E3-DHA-N: ApoE3 mice, DHA treatment, non-lipid raft fraction; E4CN, E4-CON-N: ApoE4 control mice, non-lipid raft fraction; E4DN, E4-DHA-N: ApoE4 mice, DHA treatment, non-lipid raft fraction. CL, cardiolipin; TAG, triacylglycerol; DAG, diacylglycerol; PC, phosphatidylcholine; PS, phosphatidylserine; GluCer, glucosylceramide; PI, phosphatidylinositol; CE, cholesteryl ester; SM, sphingomyelin; Cer, ceramide; PA, phosphatidic acid; FFA, free fatty acid; SL, sphingolipid; GM3, ganglioside GM3; PE, phosphatidylethanolamine; GalCer, galactosylceramide; LacCer, lactosylceramide; AC, acylcarnitine; LPI, lysophosphatidylinositol; LPE, lysophosphatidylethanolamine; LPS, lysophosphatidylserine; Sph, sphingosine; LPA, lysophosphatidic acid; LPC, lysophosphatidylcholine; PG, phosphatidylglycerol; Cho, choline

Based on the lipidomic results, differential lipids among groups were further identified (Table S3-S4). In the absence of DHA intervention, we analyzed lipid differences between lipid rafts and non-lipid rafts, as well as between ApoE3-CON and ApoE4-CON mice. The major different lipids were CL, triacylglycerol (TAG) and diacylglycerols (DAG) in ApoE3-CON group, whereas TAG, PC and DAG in ApoE4-CON mice. The two genotypes shared partial upregulated lipid species in non-lipid raft fractions, and 5 ceramide subtypes were consistently downregulated in non-lipid rafts across control groups (*P* < 0.05). Genotype‑dependent comparisons showed that lipid rafts differential lipids were mainly PCs and sulfatides (SL), with SL d18:1/20:0 as the most significantly up-regulated in ApoE4-CON mice (*P* < 0.05, FC = 1.46). In non-lipid rafts, PC 40:0 was the most up-regulated (*P* < 0.05, FC = 5.84), while Cer d18:1/24:1 was the most down-regulated lipid (*P* < 0.05, FC = 0.09) (Fig. 4a-c).

In lipid rafts, the top 8 down-regulated lipids in ApoE4-DHA *vs.* ApoE3-DHA were TAGs and SMs, whereas the top 2 up-regulated lipids were PC O-40:6 (*P* < 0.05, FC = 1.25) and PC O-40:7 (*P* < 0.05, FC = 1.41). In the non-lipid rafts, genotype‑related alterations included up-regulated PS 32:0 (*P* < 0.05, FC = 2.14), and down-regulated DAG 36:4(16:0/20:4), NeuAcHex2Cer 40:1;O2, PC O-34:2(O-16:1_18:1) and PA 38:5(18:1_20:4) (*P* < 0.05, FC < 1) (Fig. 4d-f).

In ApoE3 mice, DHA intervention increased PEs, CEs, PIs, DAGs and NeuAcHex2Cers and decreased bis (monoacylglycerol) phosphates (BMPs), TAGs and PCs in lipid rafts, with PE O-36:6 (O-16:1_20:5) the most up-regulated (*P* < 0.05, FC = 24.05) and BMP 38:6 (18:2_20:4) the most down-regulated (*P* < 0.05, FC = 0.16). DHA also extensively remodeled glycerophospholipid, neutral lipid and glycosphingolipid profiles in ApoE3 non-lipid rafts, with PE O-36:6 (*P* < 0.05, FC = 5.99) and TAG 54:4 (20:4) (*P* < 0.05, FC = 0.33) serving as the most altered lipid species. In ApoE4 mice, DHA treatment increased PEs, CEs, DAGs, PCs and CLs and decreased BMPs, TAGs, and some PCs in lipid rafts, with PE O-36:6 (O-16:1_20:5) (*P* < 0.05, FC = 16.53) and BMP 38:6 (18:2_20:4) (*P* < 0.05, FC = 0.12) were the pronounced changes. In non-lipid rafts of ApoE4 mice, DHA regulated PCs, PSs, PEs, DAGs, PIs and PGs, with PI 38:5 (18:0_20:5) the most up-regulated (*P* < 0.05, FC = 6.29) and PC O-40:5 (O-20:1_20:4) the most down-regulated (*P* < 0.05, FC = 0.23) (Fig. 4g-i).

In summary, distinct sets of differential lipids were detected between lipid-raft and non-raft fractions in ApoE3 and ApoE4 control mice, and 12-month DHA intervention triggered genotype-specific lipid alterations in both compartments, with PE O-36:6 being a prominently up-regulated lipid in both ApoE3 and ApoE4 mice.

### DHA modulates TAG, Cer and SL metabolism in a genotype- and time-dependent manner

**Fig. 5:**
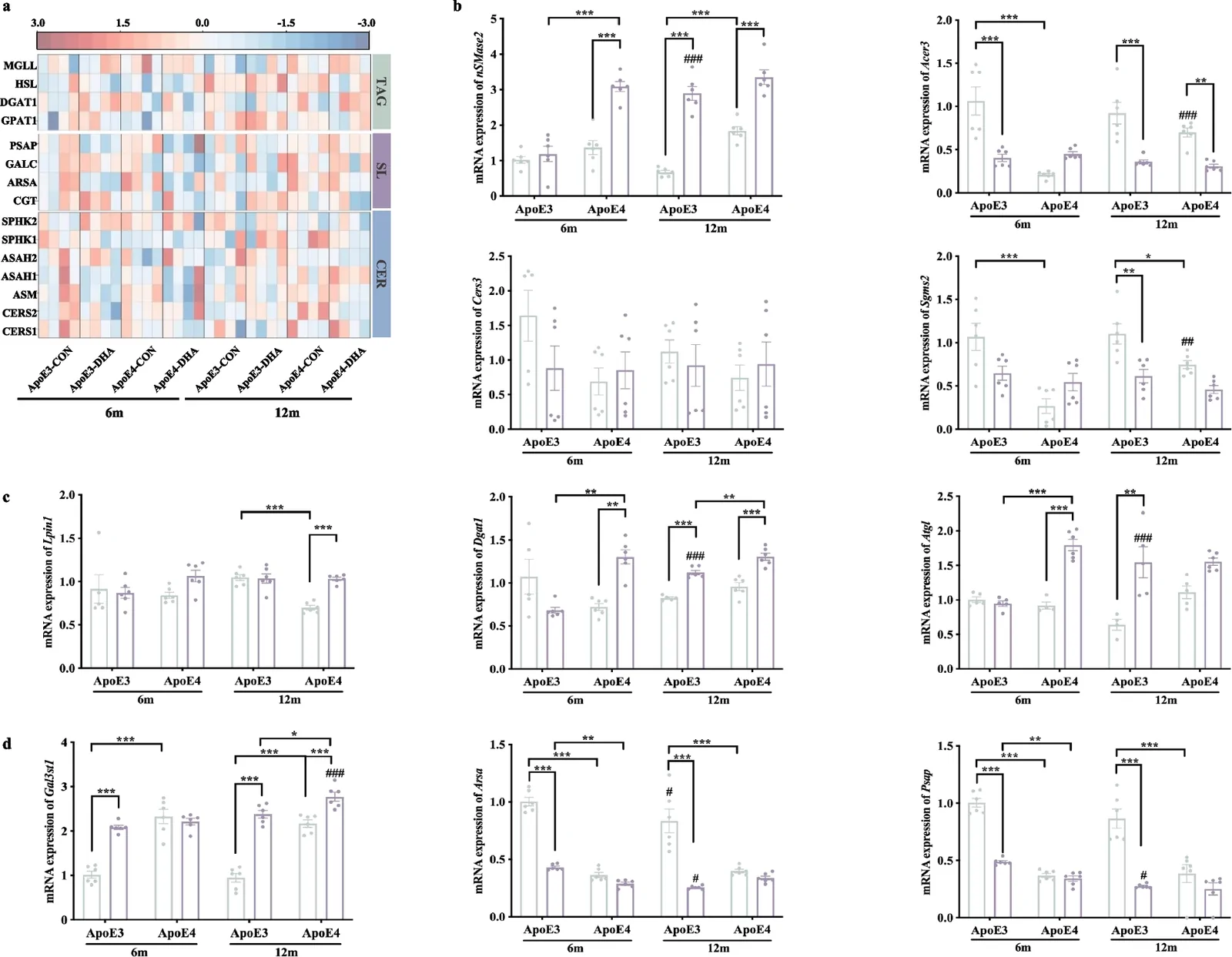
Proteins and genes expressions of TAG, SL, and CER in mice. **a** Heatmap representation of protein expression levels associated with the metabolism of TAG, SL, and CER (*N* = 4). **b** Expression of genes related to Cer metabolism (*nSMase2, Cers3, Acer3, Sgms2*) in mice (*N* = 6). **c** Expression of genes related to TAG metabolism (*Lpin1, Dgat1, Atgl*) in mice (*N* = 6). **d** Expression levels of SL metabolism-related genes (*Gal3st1, Arsa, Psap*) (*N* = 6). Cer, ceramide; TAG, triacylglycerol; SL, sphingolipid; MGLL, monoglyceride lipase; HSL, hormone-sensitive lipase; GPAT1, glycerol-3-phosphate acyltransferase 1; GALC, galactosylceramidase; CGT, ceramide galactosyltransferase; SPHK, sphingosine kinase; ASAH, acid ceramidase; ASM, acid sphingomyelinase; nSMase2, neutral sphingomyelinase 2; Cers3, ceramide synthase 3; Acer3, alkaline ceramidase 3; Sgms2, sphingomyelin synthase 2; Lpin1, lipin 1; Dgat1, diacylglycerol acyltransferase 1; Atgl, adipose triglyceride lipase; Gal3st1, galactose-3-O-sulfotransferase 1; Arsa, arylsulfatase A; Psap, prosaposin. *N* = 6 per group; mean ± SEM; Statistical significance: *** *P* < 0.001, ** *P* < 0.01, * *P* < 0.05; ^###^ *P* < 0.001, ^##^ *P* < 0.01, ^#^ *P* < 0.05 *vs.* the corresponding group after 6-month intervention

Expression levels of key proteins and genes related to TAG, Cer and SL metabolism were measured to analyze the alterations induced by ApoE genotype and DHA intervention (Table S5, Table S6). Ceramide synthase 1 (CERS1), CERS2 and acid sphingomyelinase (ASM), which participate in Cer synthesis, were reduced in 6-month DHA-treated ApoE3 mice. Acid ceramidase (ASAH1), neutral ceramidase (ASAH2), sphingosine kinase 1 (SPHK1) and SPHK2 regulate Cer breakdown. SPHK1 expression was low at 6 months but increased by 12 months. Ceramide glucosyltransferase (CGT) contributes to SL synthesis, whereas arylsulfatase A (ARSA), galactocerbrosidase (GALC), and prosaposin (PSAP) are SL hydrolysis-related enzymes. Glycerol-3-phosphate acyltransferase 1 (GPAT1) and diacylglycerol O-acyltransferase 1 (DGAT1) catalyze TAG synthesis, while hormone-sensitive lipase (HSL) and monoglyceride lipase (MGLL) mediate TAG hydrolysis. Six months of DHA intervention increased GPAT1 expression; HSL was lower at 6 months but higher after 12 months of intervention (Fig. 5a).

DHA treatment elevated cortical neutral sphingomyelinase 2* (nSMase2)* mRNA expression levels in ApoE4 mice at two interventional time points, and in ApoE3 mice, this change was only observed after 12 months intervention. In ApoE3-DHA mice, *nSMase2* mRNA expression increased with age, and a significant ApoE genotype × diet interaction was identified. ApoE4-CON mice showed lower cortical alkaline ceramidase 3 *(Acer3)* mRNA expression level than ApoE3 mice. DHA treatment down-regulated *Acer3* mRNA expression in both ApoE3 and ApoE4 mice after 12 months of intervention, while *Acer3* mRNA expression in ApoE4 mice increased with aging. An interaction of genotype × diet was observed at the 6-month interventional time point. ApoE4-CON mice exhibited lower cortical sphingomyelin synthase 2* (Sgms2)* mRNA expression level than ApoE3-CON mice. DHA treatment down-regulated *Sgms2* mRNA expression in ApoE3 mice after 12 months of intervention. *Sgms2* mRNA expression showed an age‑dependent increase, and a genotype × diet interaction was observed after 6 months of intervention (Fig. 5b, Table S1−5, *P* < 0.05).

ApoE4-CON mice exhibited lower cortical *Lpin1* mRNA expression than ApoE3 controls, 12 months of DHA intervention increased *Lpin1* mRNA expression in ApoE4 mice. An ApoE genotype-diet interaction was detected at 12 months. Cortical *Dgat1* mRNA expression was similar in ApoE3 and ApoE4 mice, DHA dietary intervention increased it at 12 months, with a genotype-diet interaction observed at 6 months. Six months of DHA intervention increased cortical adipose triglyceride lipase (*Atg1)* mRNA expression in ApoE4 mice, but in ApoE3 mice at 12 months. With aging, the cortical *Atg1* mRNA expression increased in ApoE3-DHA mice, and an interaction was detected at 6 months (Fig. 5c, Table S1−5, *P* < 0.05).

DHA intervention induced differential regulation of sphingolipid catabolic and synthetic genes between ApoE genotypes. ApoE4-CON mice showed higher cortical galactose-3-O-gulfotransferase 1 *(Gal3st1)* mRNA expression than ApoE3-CON mice. Six months of DHA intervention increased *Gal3st1* mRNA expression in ApoE3 mice, and 12 months of intervention up-regulated *Gal3st1* in both genotypes. Cortical *Gal3st1* mRNA expression exhibited an age-related increase in ApoE4 mice, and a genotype-diet interaction was detected. In contrast, ApoE4-CON mice had lower cortical *Arsa* and *Psap* mRNA expression than ApoE3-CON mice, DHA treatment down-regulated *Arsa* and *Psap* mRNA expression in ApoE3 mice. Genotype × diet interactions for *Arsa* and *Psap* mRNA expression were also observed (Fig. 5d, Table S1−5, *P* < 0.05).

In summary, DHA exerted time- and genotype-dependent regulatory effects on key genes and proteins involved in ceramide, sphingolipid and TAG metabolism, with multiple significant genotype-diet interactions identified.

### Temporal and genotypic specificity of lipid raft proteomic responses to DHA supplementation

**Fig. 6:**
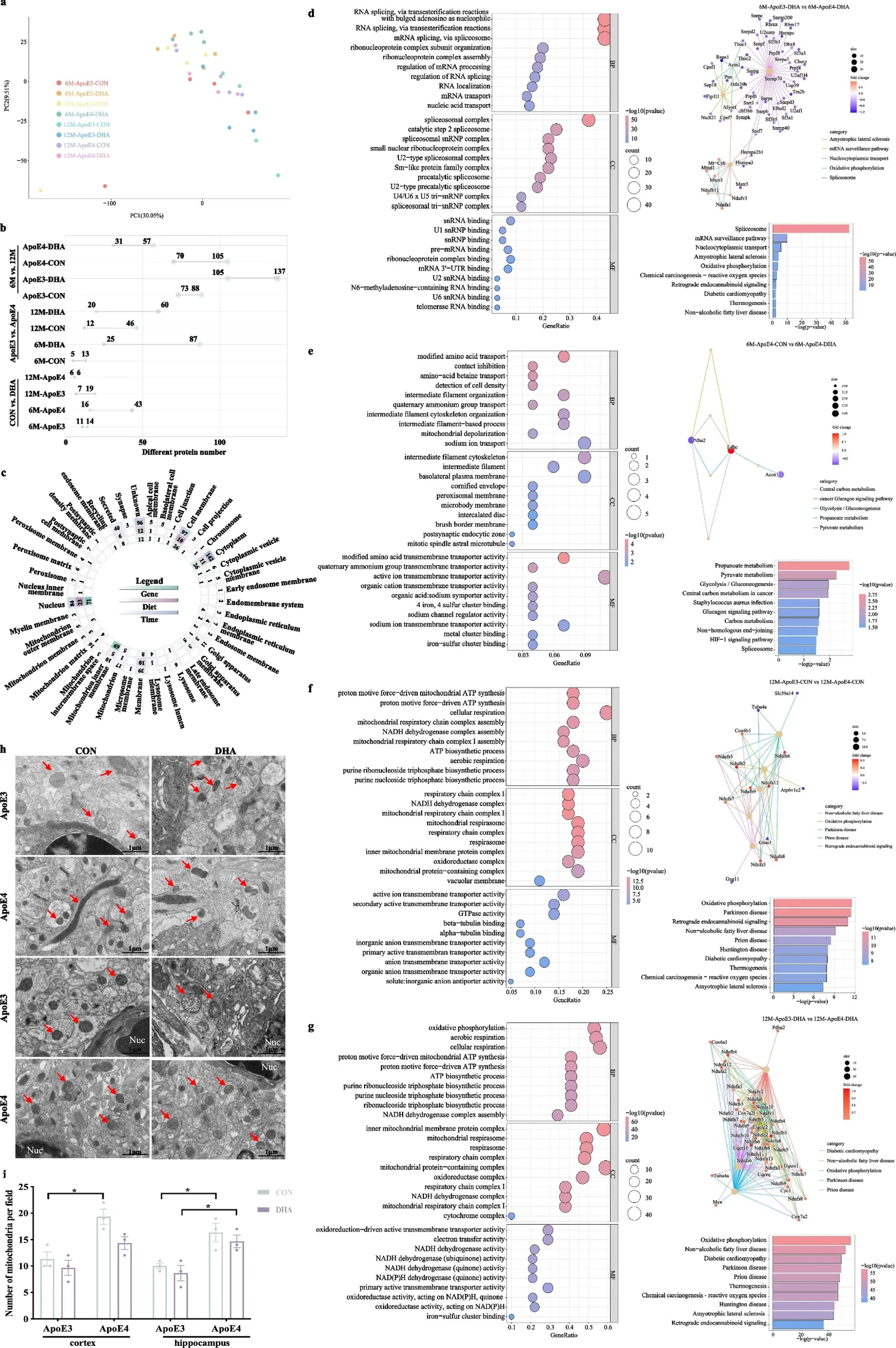
Proteomic Profiling of Brain Lipid Rafts, Functional Enrichment Analysis, and Mitochondrial Ultrastructure. **a** Principal component analysis (PCA) of proteomic profiles from mouse brain lipid raft fractions. **b** Bar chart showing the number of upregulated and downregulated differentially expressed proteins among intergroups. **c** Circular heatmap integrating subcellular localization patterns of proteins differentially regulated by genotype, diet, and intervention duration. **d**-**f** Enrichment analysis of Gene Ontology (GO) terms or Kyoto Encyclopedia of Genes and Genomes (KEGG) pathways between groups. **g** Transmission electron microscopy (TEM) images of mitochondria (red arrows) in the cerebral cortex and hippocampus of mice, with “Nuc” indicating nucleus. h: Quantitative analysis of the number of mitochondria per field in the cerebral cortex and hippocampus, *N* = 3, mean ± SEM. Scale bar = 1 μm; Statistical significance: * *P* < 0.05

We performed proteomic profiling of lipid raft proteins followed by enrichment analysis to screen for differentially enriched signaling pathways. Samples from the mice at 6-month interventional time point clustered in the negative PC1 region, while the samples from mice at 12-month interventional time point clustered in the positive region, indicating that intervention duration was a key driver of variance. ApoE genotype and DHA treatment also contributed to proteomic differences, and their interactive effects are illustrated in Fig. 6a-b. DHA enhanced protein differences in ApoE3 mice, but reduced them in ApoE4 mice, and overall protein divergence increased with age. The subcellular localization of deferentially expressed proteins is summarized in Fig. 6c. Genotype-related proteins mainly were localized to the nucleus, inner mitochondrial membrane and cell membrane; diet-related proteins were enriched in the nucleus, cell membrane and cytoplasm; while duration-related differential proteins were predominantly found in the cytoplasm, cell membrane and nucleus.

Significant differences in RNA processing and post‑transcriptional regulation were detected between 6 M‑ApoE3‑DHA and 6 M‑ApoE4‑DHA groups. The protein–protein interaction (PPI) networks and KEGG enrichment highlighted the spliceosome as central node, with additional involvement of oxidative phosphorylation and the mRNA surveillance pathways (Fig. 6d). For 6 M-ApoE4-CON *vs.* 6 M-ApoE4-DHA, enriched biological processes (BP) included amino acid transport, cell proliferation and cytoskeletal remodeling, cell component (CC) were mainly cytoskeleton and membrane, and molecular function (MF) involved transporter activity and molecular binding. PPI and KEGG analyses highlighted metabolic pathways, including glycolysis, pyruvate, and propanoate metabolism, central carbon metabolism, and HIF-1 signaling (Fig. 6e). For 12 M-ApoE3 *vs.* ApoE4, BP terms were enriched for mitochondrial energy metabolism, especially the respiratory chain, and CC terms were concentrated in core mitochondrial components, and MF terms involved transport, cytoskeletal interactions (CON) and mitochondrial metabolic activity (DHA). PPI and KEGG analyses emphasized oxidative phosphorylation, and its links to Parkinson’s disease, non-alcoholic fatty liver disease (NAFLD) and the broader associations between mitochondrial dysfunction, neurodegenerative disease and metabolic disorders (Fig. 6f-g).

Collectively, proteomic profiling revealed intervention duration as the primary source of variation, together with ApoE genotype-dependent DHA effects; enrichment analyses highlighted spliceosome, glycolytic metabolism and mitochondrial oxidative phosphorylation as key altered pathways.

### Neuronal mitochondrial ultrastructure in cortex and hippocampus

Finally, TEM was used to analyze the inter-group differences in mitochondrial ultrastructure and quantity. As shown in Fig. 6i-j, ApoE4-CON mice had more mitochondria in cortical and hippocampal neurons than ApoE3-CON mice, indicating that pathological mitochondrial hyperplasia occurs as an adaptive response to cellular damage. ApoE3-CON mice exhibited more pronounced mitochondrial matrix swelling, with larger, rounded mitochondria showing obvious morphological abnormalities, reduced matrix electron density and shorter, sparser mitochondrial cristae. On the other hand, ApoE4-CON mice displayed relatively well-preserved neuronal mitochondrial ultrastructure, characterized by clearly defined double membranes, intact internal cristae, and smooth outer membrane contours. Numerous electron-dense deposits were also visible within the mitochondrial matrix, potentially representing degradation products of mitochondrial components. In summary, assessment of mitochondrial ultrastructure confirmed pathological mitochondrial hyperplasia in the neurons of ApoE4-CON mice.

## Discussion

Epidemiological studies indicate that ApoE4-associated AD risk is markedly higher in females. Compared with male ε4 carriers, female ε4 carriers exhibit a higher conversion rate from mild cognitive impairment to AD, with a 2 ~ threefold increased disease risk [19]. Consistently, animal studies confirm that female ApoE4 mice display the most severe Aβ deposition, neuroinflammation, and cognitive deficits. Male ApoE4 mice exhibit a pathological burden comparable to that of female ApoE3 mice; the combination of ApoE4 and female sex confers the highest AD risk [20]. Additionally, sex differences exist in DHA sensitivity: aged female ApoE4 mice display an age‑dependent reduction in brain DHA and substantial fluctuations in lipid phenotypes despite identical dietary DHA intake, whereas male ApoE4 mice maintain relatively stable cerebral DHA homeostasis with only modest lipid responses [21]. Since ApoE4 and female are well-established AD risk factors, female mice were selected for the present study. We acknowledge that the exclusive use of female mice limits generalizability to males; future studies should include male mice to investigate sex‑dependent effects.

Hepatic lipid accumulation is a key feature of systemic metabolic dysregulation. ApoE4 exhibits hypolipidated properties and tends to form non-lipidated aggregates, which impair lipid turnover, and promote lipid droplet accumulation [22]. However, some studies have reported that the human ApoE ε4 allele may confer protection against NAFLD, whereas ε3 appears to promote hepatic steatosis and steatohepatitis. After 7 months on a high-fat, high-sucrose diet, ApoE3 male mice exhibited higher liver weights and more pronounced steatosis than ApoE4 males [23]. In our study, both ApoE3 and ApoE4 mice developed hepatic lipid deposition following 12-month intervention compared to 6-month controls, with ApoE4 mice exhibiting more severe accumulation. This pattern indicates ApoE genotype- and age-dependent exacerbation of lipid metabolism disorder. Consistent with our previous work [24], DHA intervention attenuated hepatic lipid deposition in both genotypes, demonstrating its potential to counteract ApoE genotype and age related hepatic lipid metabolism dysfunction.

Although spatial behavioral impairment was undetectable, 14‑month‑old ApoE4/4 control mice displayed changes in key AD‑related pathological markers, including impaired amyloid and tau metabolism, pronounced overactivation of astrocytes and microglia, neuronal loss in the hippocampus and cortex, and mitochondrial ultrastructural damage. These changes reflect early neuronal vulnerability that precedes measurable cognitive decline. Consistent with previous reports on this humanized ApoE knock-in strain, female ApoE4/4 mice at this age do not develop robust spatial learning and memory deficits in the Morris water maze, despite exhibiting multiple early AD-relevant molecular, lipid, and glial pathological alterations [25, 26]. Our behavioral test battery is relatively limited. Future studies will incorporate additional cognitive paradigms such as the Y‑maze and novel‑object recognition test to enable a more comprehensive cognitive assessment.

GFAP upregulation is a hallmark of astrocyte activation. Previous study shown that ApoE4 mice displayed more numerous astrocytes with larger cell bodies and more processes than ApoE3 mice [27]. Long-term DHA dampens astrocyte reactivity and slows the escalation of ApoE4-driven neuroinflammation. Under physiological conditions, microglia display a ramified morphology but transform into an amoeboid shape in AD, acquiring a chronic pro-inflammatory phenotype that drives neuroinflammation [28]. DHA possesses anti-inflammatory properties by altering membrane phospholipid composition and perturbing lipid rafts [28]. We found age-dependent amplification of microglial activation in the cortex of ApoE4 mice. 12-month DHA intervention reduced cortical Iba1 expression in ApoE4 mice, suggesting a time- and ApoE-genotype dependent effect of DHA on alleviating neuroinflammation.

ApoE4 mice and ApoE4 carriers show higher Aβ oligomerization, deposition and burden than ApoE3 counterparts [29, 30]. DHA enhances Aβ clearance and modulates Aβ aggregation by shifting Aβ towards to the metabolism of less toxic fibrillar oligomers in animals [31, 32]. DHA intervention reduced APP expression in cortex of ApoE3 mice, suggesting a potential regulating effect of DHA on upstream Aβ precursor availability in mice with the ApoE3 genotype. ApoE4 correlates with higher tau levels [33], and DHA attenuates tau hyperphosphorylation in Tau-overexpressing mice [34]. This study detected no abnormal tau phosphorylation in ApoE4 mice nor DHA effects on tau, consistent with a model in which our intervention window precedes overt Aβ/tau pathology and instead primarily targets earlier metabolic. Elevated phosphorylated tau was observed in ApoE3 mice following 6-month DHA intervention. ApoE3 mice lack endogenous amyloid pathology and maintain intact basal membrane lipid homeostasis. Exogenous DHA profoundly remodels the sphingolipid and glycerophospholipid composition of lipid rafts and alters membrane fluidity, which transiently activates membrane-bound kinases such as GSK3β and CDK5 and leading to moderately upregulated tau phosphorylation [35]. This transient tau phosphorylation likely represents a compensatory response to disrupted membrane homeostasis rather than neurodegenerative pathology. Long-term DHA corrected membrane lipid dysregulation, suppressed pathological kinases, and conferred neuroprotection.

DHA primarily localizes to lipid rafts, and non-lipid rafts are mainly SFA (~90%), with DHA and AA < 1%. PE is rich in lipid rafts, while PS is rich in non-lipid rafts. CL, essential for mitochondrial function and neurodegeneration, showed low raft abundance [36, 37]. ApoE4 mice exhibited reduced CL in non-lipid rafts, indicating mitochondrial impairment. Cholesterol was predominantly raft-localized regardless of genotype or dietary [38]. Remarkably, ApoE4 mice have almost no cholesterol in non-lipid rafts, whereas ApoE3 mice exhibited non-raft cholesterol levels equivalent to roughly half of their raft cholesterol. Among glycosphingolipids, only GluCer displayed clear segregation between raft and non-raft compartments. Together, these data indicate that ApoE4 brains have more sphingolipid-rich, cholesterol-dominated rafts and a more SFA-enriched, cholesterol-poor non-raft environment, a pattern that is likely to stiffen membranes and alter signaling.

Lipid component varies across species, tissues, organelles, and membrane leaflets [39]. The “lipid raft aging hypothesis” posits that age-related lipid alterations in lipid rafts modify their characteristics and contribute to AD [40]. Aging affects cerebral lipid rafts, and AD genotypes synergize with aging to accelerate these changes, as APP/PS1 mice exhibit earlier and severe lipid raft alterations [17]. This study found that non-lipid rafts contain less cholesterol, more GPs, and similar sphingolipids proportions. DHA modulate lipid raft phospholipid composition by competing with rigid cholesterol and sphingolipids. ApoE4 mice had higher SL and lower TAG in lipid rafts, and lower Cer in non-lipid rafts than ApoE3 mice. This pattern is consistent with a shift of neutral lipids away from ApoE4 rafts and a reciprocal accumulation of complex sphingolipids, potentially rendering rafts more rigid and signal-competent.

Our lipidomic analysis identified increased levels of the ether phospholipids PC O-40:6 and PC O-40:7 in brain lipid rafts from ApoE4-DHA mice versus ApoE3-DHA mice. Across genotypes, DHA supplementation elevated PE O-36:6 (O-16:1_20:5) in lipid rafts. Plasmalogens, which are highly in synaptic membranes, myelin sheaths, and lipid rafts, support membranes integrity, antioxidant defense, synaptic function, and microglial homeostasis. Plasmalogen deficiency, observed in AD patients and correlated with disease severity, disrupts the lipid raft microenvironment, promotes Aβ generation, neuroinflammation, and tau hyperphosphorylation, triggering a pathological vicious cycle [41]. Plasmalogen status may mediate ApoE-related dementia risk, and high plasmalogen biosynthesis confers dementia protection regardless of age or ApoE background via the regulation of cholesterol homeostasis [42].

DHA upregulated *Lpin1* and *Atgl* in ApoE4 mice at 6 months, and *Lpin1* and *Dgat1* at 12 months, suggesting enhanced TAG turnover and potential redistribution of TAG from rafts to non-raft. Cer accumulation is a feature of aging and neurodegeneration, where Cer can drive axonal degeneration and neuronal apoptosis [43]. 6-month DHA intervention reduced *Acer3* and *Sgms2*, suppressing Cer degradation, especially in ApoE4 mice, favoring Cer accumulation and its potential relocalisation into lipid rafts. PS19 transgenic AD mice exhibit elevated Cer and nSMase2 activity [44, 45]. ApoE4-CON mice showed higher *Gal3st1* but lower *Arsa* and *Psap*, with elevated SL in lipid rafts. Combined with water maze findings, we propose that ApoE4 mice maintain a compensatory state before symptom onset, augmenting cerebral SL reserves via enhanced synthesis and reduced degradation as a protective response against subsequent SL loss. DHA intervention enhanced SL synthesis and reduced decomposition in ApoE3 mice lipid rafts, which may contribute to long-term resilience against AD-related lipid imbalance.

The subcellular localization of proteins reflects the systemic impact of aging on cellular functions, with common differential proteins predominantly localized to the mitochondrial inner membrane (Table S7). After DHA treatment in mice, spliceosomes components emerged as central and were linked to oxidative phosphorylation, suggesting that ApoE genotype modulates cellular function by affecting RNA processing and energy metabolism. In ApoE4 mice, DHA may improve metabolic imbalance caused by ApoE4 genotype through regulating basal metabolic processes. At 12 months, differences in mitochondrial energy metabolism pathways between ApoE genotypes were observed, overlapping with disease pathways such as Parkinson’s disease. These findings support that long-term ApoE4 expression disrupts mitochondrial bioenergetics, and DHA partially normalizes these changes, positioning mitochondrial dysfunction as a mechanistic bridge between metabolic disorders and neurodegenerative disease. The ultrastructural differences of mitochondria further underscore genotype-specific patterns of mitochondrial damage. ApoE4-CON mice exhibited an increased number of neuronal mitochondria. ApoE3-CON mice displayed more overt structural damage, indicating distinct models of mitochondrial injury between ApoE isoforms. Although mitochondrial morphology appeared relatively preserved in ApoE4-CON mice, defects in mitochondrial quality control may still compromise function, in agreement with proteomic evidence.

This study has several limitations. Firstly, the animal model did not reach the elderly stage of mice, and the AD phenotype was not very pronounced. Secondly, this study utilized fish oil as the source of DHA, which has a significant disadvantage over phospholipid krill oil. Additionally, individual-level precise DHA intake data were unavailable, which may lead to unquantifiable experimental variations. Furthermore, the results of the animal experiments lack human validation and should be further validated using in vitro cell models. Notably, the absence of region-specific omics analysis of lipid rafts in the cortex and hippocampus currently limits further elucidation of the direct association between DHA-induced lipid raft remodeling and regional pathological vulnerability in AD. Finally, the coupling mechanism between differential lipids and lipid raft abnormalities has not been elucidated, and multi omics integration and analysis of regulatory pathways are needed. Future studies should address these shortcomings to consolidate the current conclusions.

## Conclusion

This study demonstrates that the ApoE4 genotype promotes age-associated hepatic lipid accumulation and neuroinflammatory changes. Lipidomic and proteomic profiling revealed distinct alterations in lipid rafts and non-rafts, with increased sphingolipid accumulation, reduced cardiolipin, and mitochondrial function damage particularly in ApoE4 mice. Notably, DHA reduced hepatic lipid deposition, restructured raft and non-raft lipid composition, and partially restored mitochondrial and glial homeostasis. Together, these findings provide mechanistic evidence that long-term DHA supplementation can modulate ApoE4-driven disruptions in lipid and energy metabolism across liver and brain, and may offer a strategy to delay progression toward neurodegenerative disease.

## Material and methods

### Animals

Eight-week-old, healthy female ApoE3/3 and ApoE4/4 mice (n = 37) were purchased from Cyagen Biotechnology (Suzhou) Co., Ltd. After a 2-week acclimatization with free access to water and feed, the mice were randomly divided into 4 groups based on body weight: (1) ApoE3-CON: ApoE3/3 mice on a control diet; (2) ApoE3-DHA: ApoE3/3 mice on a DHA-supplemented diet; (3) ApoE4-CON: ApoE4/4 mice on a control diet; (4) ApoE4-DHA: ApoE 4/4 mice on a DHA-supplemented diet. Body weight and daily feed intake were recorded weekly, and detailed sample size information is provided in Table S8. A schematic is shown in Fig. 1a (Supplementary note). The experiment feed was obtained from SYSE Biotechnology (Changzhou) Co., Ltd, and DHA was derived from fish oil powder provided by Royal DSM (50–1526-9 Netherlands) (Table S9).

### Behavioral tests

After 6 or 12 months of dietary intervention, the Morris water maze test was conducted following the established protocols [46].

### Biological sample collection

After the behavioral tests, mice were anesthetized and subsequently euthanized, followed by immediate dissection of liver and brain tissue. The liver and the left hemisphere were immersed in 4% paraformaldehyde for fixation at room temperature prior to subsequent immunohistochemical analysis. The hippocampus and cortex were rapidly isolated from the remaining brain tissue and stored at −80℃.

### Nissl staining

Sections were sequentially immersed in xylene I for 20 min, xylene II for 20 min, absolute ethanol I for 10 min, absolute ethanol II for 10 min, 95% ethanol for 5 min, 90% ethanol for 5 min, 80% ethanol for 5 min, and 70% ethanol for 5 min, followed by rinsing with distilled water. Sections were incubated with 1% cresyl violet staining solution at room temperature for 45 min, and then rinsed with water. Sections were differentiated in 70% ethanol for 2 min until most excess staining was removed. Sections were placed in 70% ethanol for 2 min, 80% ethanol for 2 min, 95% ethanol for 2 min, absolute ethanol I for 5 min, absolute ethanol II for 5 min, xylene I for 10 min, and xylene II for 10 min for dehydration and clearing. After brief air-drying, sections were mounted with neutral balsam.

### Hematoxylin–eosin staining

The slices were dewaxed, then stained with 1% hematoxylin for 5 min. Next, the slices were differentiated using 1% hydrochloric acid in alcohol, then rinsed and soaked in 1% ammonia. After that, they were stained with eosin. Finally, the slices were treated sequentially with ethanol, anhydrous ethanol, and xylene, and were sealed with neutral gum afterward.

### Histological measurement

The expression of cerebral anti-glial fibrillary acidic protein (GFAP, ab66217, Abcam, UK) and hepatic anti-ionized calcium binding adaptor molecule 1 (Iba1, ET1705-78, HuaBio, China) was examined via histological measurement as previously described [46].

### Enzyme Linked Immunosorbent Assay (ELISA) and western blotting

Cortical tissue samples were mixed and homogenized by grinding on ice. After centrifugation at 4 ℃ and 5,000 × g for 10 min, the supernatants were collected for biochemical assays. Protein concentration was measured using a bicinchoninic acid protein quantification kit (BCA01, Dingguo Changsheng, China). The levels of ApoE (RK00231, ABclonal, China), Aβ_1–40_ (E-EL-M3009, Elabscience, China), and Aβ_1–42_ (E-EL-M3010, Elabscience, China) were quantified following the instructions.

Cortical protein lysates were electrophoresed on Tris-tricine gradient gels as described previously [46]. The antibodies are listed in Table S10. Raw blots and quantitative analysis values are provided in Table S11 and Fig. S1.

### Quantitative real-time PCR (RT-qPCR)

RNA was isolated using the Fast Pure Cell/Tissue Total RNA Isolation Kit (Vazyme, China). Reverse transcription and target gene amplification assays were performed as described previously [46]. *GAPDH* was used as the housekeeping gene, and the PCR primers used in this experiment are listed in Table S12. Relative gene expression levels were calculated using the 2^−ΔΔCt^ method.

### Transmission Electron Microscopy (TEM)

Mice brains were perfused‑fixed, dehydrated, embedded and polymerized. 70 nm ultrathin sections were double‑stained and imaged by transmission electron microscopy. Full experimental procedures are described in Supplementary Methods.

### Isolation of lipid raft and non-lipid raft fractions

Minute™ Lipid Raft Isolation Kit (LR-039, Invent, USA) and Minute™ High Efficiency Protein Precipitation Kit (WA-006, Invent, USA) were used for the separation of cerebellum total lipid raft and non-lipid raft components following the manufacturer’s instructions. Lipids were extracted from lipid raft and non-lipid raft fractions using a modified Bligh and Dyer’s method as described previously [47].

### Lipidomic analysis

Lipidomic analyses were conducted at LipidALL Technologies using a Shimadzu Nexera 20-AD HPLC coupled with Sciex QTRAP 6500 PLUS as reported previously [48]. Glycerol lipids were quantified using a modified version of reverse phase HPLC/MRM [49]. Free cholesterols and CE were analyzed under atmospheric pressure chemical ionization (APCI) mode on a Jasper HPLC coupled to Sciex 4500 MD, internal standards were listed in Table S13 [50].

### Proteomics analysis

Peptides were prepared via precipitation, denaturation, reduction-alkylation, two-step tryptic digestion and SOLAμ SPE desalting. Samples underwent timsTOF Pro-based LC–MS/MS with DDA library building and DIA acquisition. Raw data of three batches were analyzed by DIA-NN v1.8.1. Full experimental procedures are described in Supplementary Methods.

### Statistical analysis

Data were expressed as mean ± standard error of the mean (SEM). Data meeting normal distribution and homogeneity of variance assumptions were compared using two-way Analysis of Variance, with post hoc multiple comparisons conducted via Tukey’s method. For data that did not conform to a normal distribution or exhibited unequal variances after transformation, non-parametric tests were employed. Statistical significance was set at *P* < 0.05.

## Supplementary Information

Supplementary Material 1.

## Data Availability

The datasets used and/or analyzed during the current study are available from the corresponding author on reasonable request.
