# Supplementary material for: Docosahexaenoic acid remodels lipid composition of lipid rafts and mitochondrial function in ApoE4/4 mouse brain: a multi-omic investigation
Source: Mol Biomed. 2026 Oct 15;7:212. doi: 10.1186/s43556-026-00618-x (PMC13672281; doi:10.1186/s43556-026-00618-x)
Supplement: Supplementary file 1 [file 43556_2026_618_MOESM1_ESM.docx]

**Supplementary material contents**

**Supplementary note of Fig.1a	Page 2**

**Supplementary Methods	Page 2-6**

**Supplementary Table 1	Page 6-11**

**Supplementary Table 2	Page 12-14**

**Supplementary Table 3	Page 15-20**

**Supplementary Table 4	Page 21-36**

**Supplementary Table 5	Page 37-39**

**Supplementary Table 6	Page 40-41**

**Supplementary Table 7	Page 42**

**Supplementary Table 8	Page 43-44**

**Supplementary Table 9	Page 45**

**Supplementary Table 10	Page 46**

**Supplementary Table 11	Page 47-50**

**Supplementary Table 12	Page 51-52**

**Supplementary Table 13	Page 53**

**Supplementary Figure 1	Page 54-55**

**Supplementary note of Fig.1a**

ApoE3 and ApoE4 mice received dietary DHA intervention for 6 or 12 months. Cognitive‑behavioral performance was evaluated using the Morris water maze test. Brain, liver, blood and other samples were subsequently collected. Lipid‑raft and non‑lipid‑raft sub‑fractions were isolated from cerebellum. Lipidomic, proteomics, qPCR, Western blot and transmission electron microscopy (TEM) were jointly applied to dissect the DHA‑mediated, ApoE-genotype‑dependent regulation on brain lipid‑raft lipid remodelling, molecular pathway alterations and mitochondrial ultrastructure.

**Supplementary Methods**

***Transmission electron microscopy (TEM)***

(1) Perfusion, tissue harvesting and fixation: After 12 h of fasting, mice were anesthetized by intraperitoneal injection. The thoracic cavity was opened, and the heart was perfused with normal saline. Once the liver turned pale, perfusion was continued with 4% paraformaldehyde. Brain tissues were rapidly dissected, and the cortex and hippocampus were isolated. Tissues were cut into small blocks (1 mm × 1 mm × 1 mm) and fixed overnight in 2.5% glutaraldehyde at 4 °C. Samples were washed three times with 0.1 M phosphate buffer (PB), 15 min per wash. Subsequently, tissues were post-fixed in 1% osmium tetroxide for 2-3 h, followed by three washes with 0.1 M PB (15 min for each time);

(2) Dehydration: Samples were dehydrated at 4 °C in 50% ethanol (15 min), 70% ethanol (15 min), 90% ethanol (15 min), 90% ethanol-90% acetone (1:1, 15 min), and 90% acetone (15 min). At room temperature, samples were further dehydrated with 100% acetone I (15 min), 100% acetone II (15 min), and 100% acetone III (15 min);

(3) Embedding: Samples were incubated in pure acetone-embedding medium (2:1) at room temperature for 3-4 h, then in pure acetone-embedding medium (1:2) at room temperature overnight, and finally in pure embedding medium at 37 °C for 3 h;

(4) Polymerization: Tissues were polymerized sequentially at 37 °C overnight, 45 °C for 12 h, and 60 °C for 48 h;

(5) Sectioning: Ultrathin sections of 70 nm thickness were prepared using an ultramicrotome;

(6) Staining: Sections were double-stained with 3% uranyl acetate-lead citrate for 10 min, rinsed with distilled water, and air dried. Prepared sections were observed and imaged under a transmission electron microscope.

***Proteomic***

*1 Peptide preparation*

(1) Protein precipitation: 200 μL of 100% acetone was added to each 1.5-mL centrifuge tube, and proteins were precipitated at -20 °C overnight. After centrifugation, the supernatant was discarded. The pellet was washed once with 200 μL 80% acetone and twice with 200 μL 100% acetone;

(2) Protein denaturation: 30 μL of lysis buffer (6 M urea, 2 M thiourea) was added to each tube. Samples were incubated in a thermomixer (MS-100) at 31.5 °C with shaking at 600 rpm for 30 min;

(3) Reduction: 1.6 μL of 0.2 M TCEP/100 mM ABB was added to achieve a final TCEP concentration of 10 mM. Samples were incubated at 31.5 °C, 600 rpm for 30 min;

(4) Alkylation: 1.65 μL of 0.8 M IAA/100 mM ABB was added to reach a final IAA concentration of 40 mM. Samples were incubated at 31.5 °C, 600rpm in the dark for 30 min;

(5) Digestion I: Trypsin (100 μg) was dissolved in 1 mM HCl or 50 mM HAC to a concentration of 0.5 μg/μL. 200 μL of 0.1 M ABB and 6 μL trypsin solution (3 μg trypsin) were added to each tube, and incubated at 32 °C, 600 rpm for 4 h;

(6) Digestion II: An additional 6 μL trypsin solution (3 μg trypsin) was added, and digestion proceeded at 32 °C, 600 rpm for 12 h;

(7) Termination of digestion: 25 μL of 10% TFA was added to yield a final TFA concentration of 1%, and the pH was adjusted to 2-3;

(8) Desalting: Desalting was performed using SOLAμ SPE plates (Thermo Fisher Scientific^TM^, San Jose, USA). Column activation: 200 μL MeOH, twice. Column equilibration: 200 μL 80% ACN in 0.1% TFA, twice. Column washing: 200 μL 2% ACN in 0.1% TFA, twice. After sample loading, the column was washed ten times with 200 μL 2% ACN in 0.1% TFA. Peptides were eluted twice with 100 μL 40% ACN in 0.1% TFA. Eluates were vacuum‑concentrated to dryness at 40 °C under pressure below 10 mBar. Peptide concentration was determined by A280 absorbance after reconstitution;

(9) Mass‑spectrometry injection: For library‑building DDA acquisition, peptides from all samples were pooled to generate one pooled sample containing 100 μg total peptides. For routine DIA acquisition, peptide concentration was adjusted to 0.2 μg/μL; 1 μL was injected, corresponding to 200 ng peptide load per DIA run.

*2 LC‑MS/MS analysis*

LC‑MS/MS was performed on a UHPLC system coupled to a timsTOF Pro mass spectrometer (Bruker Daltonics, Germany). DDA was used for spectral library construction, and DIA for sample acquisition. Mobile phase A: water with 0.1% formic acid; mobile phase B: acetonitrile with 0.1% formic acid. All solvents were MS‑grade. For DDA library acquisition, peptides were loaded onto a trap column (5 mm × 300 µm i.d.) at 217.5 bar, then separated on an analytical column (1.9 µm, 120 Å, 150 mm × 75 µm i.d.) at a flow rate of 300 nL/min with a 60-min gradient: 0 50 min, 5%-27% B; 50-60 min, 27%-40% B. MS parameters: PASEF MS and MS/MS m/z range 100-1700; 1/k0 range 0.6-1.6; mobility peak threshold 5000; 10 PASEF MS/MS scans per cycle; charge range 0-5; peak intensity threshold 2500 cts/s. For DIA acquisition of all samples, peptides were loaded onto the same trap‑analytical column setup with identical flow rate and 60‑min gradient. DIA‑MS parameters: PASEF MS m/z 100-1700, 1/k0 0.7-1.3, resolution 60 000, mobility peak threshold 5000; 10 PASEF MS/MS scans per cycle, charge range 0-5, peak threshold 2500 cts/s, 56 DIA windows.

*3 MS data processing*

Raw MS files from three experimental batches were processed using DIA‑NN software (version 1.8.1).

All data obtained from lipidomic detection underwent lipid concentration normalization through quantification of phosphatidylcholines (PC) content. Statistical analysis and graph generation were performed using https://jvenn.toulouse.inra.fr/app/index.html, SPSS 22.0, R 4.3.1, and GraphPad Prism 8.0 software. Raw DIA data were decoded against the DDA-derived spectral library using DIA-NN software. For proteomics analysis, searches were conducted using the UniProt-reviewed human proteome database, accounting for fixed carbamidomethylation of cysteines and variable methionine oxidations. Match-between-runs technology improved cross-sample identification consistency, with protein and precursor identifications stringently filtered at 1% false discovery rate.

**Supplementary Table 1. *P-* value of Two-way ANOVA**

1-1. Number of crossing the platform

| Intervention period | Factors | | Interactions |
| --- | --- | --- | --- |
|  | genotype | diet | genotype🞨diet |
| 6 months | *P* = 0.30 | *P* = 0.41 | *P* = 0.68 |
| 12 months | *P* = 0.46 | *P* = 0.21 | *P* = 0.87 |

1-2. Cortical ApoE, Aβ_1-40_, Aβ_1-42_ content and Aβ_1-40_/Aβ_1-42_ ratio

| Parameters | Intervention period | Factors | | Interactions |
| --- | --- | --- | --- | --- |
|  |  | genotype | diet | genotype🞨diet |
| ApoE | 6 months | *P* = 0.72 | *P* = 0.02 | *P* = 0.84 |
|  | 12 months | *P* < 0.001 | *P* < 0.001 | *P* < 0.001 |
| Aβ_1-40_ | 6 months | *P* = 0.52 | *P* = 0.07 | *P* = 0.21 |
|  | 12 months | *P* = 0.91 | *P* = 0.39 | *P* = 0.19 |
| Aβ_1-42_ | 6 months | *P* = 0.001 | *P* = 0.12 | *P* = 0.43 |
|  | 12 months | *P* = 0.10 | *P* = 0.10 | *P* = 0.09 |
| Aβ_1-40_/Aβ_1-42_ | 6 months | *P* = 0.15 | *P* = 0.47 | *P* = 0.63 |
|  | 12 months | *P* = 0.47 | *P* = 0.83 | *P* = 0.03 |

1-3. Proteins Expression levels of CAV1, BACE1, APP, LRP1, IDE, tau, p-tau, and p-tau/tau ratio

| Parameters | Intervention period | Factors | | Interactions |
| --- | --- | --- | --- | --- |
|  |  | genotype | diet | genotype🞨diet |
| BACE1 | 6 months | *P* = 0.26 | *P* = 0.58 | *P* = 0.22 |
|  | 12 months | *P* = 0.43 | *P* = 0.12 | *P* = 0.73 |
| APP | 6 months | *P* = 0.04 | *P* = 0.007 | *P* = 0.001 |
|  | 12 months | *P* = 0.06 | *P* = 0.12 | *P* = 0.22 |
| LRP1 | 6 months | *P* = 0.01 | *P* = 0.16 | *P* = 0.49 |
|  | 12 months | *P* = 0.23 | *P* = 0.49 | *P* = 0.94 |
| IDE | 6 months | *P* = 0.52 | *P* = 0.95 | *P* = 0.70 |
|  | 12 months | *P* = 0.69 | *P* = 0.13 | *P* = 0.01 |
| tau | 6 months | *P* = 0.04 | *P* = 0.35 | *P* = 0.17 |
|  | 12 months | *P* = 0.46 | *P* = 0.99 | *P* = 0.57 |
| p-tau | 6 months | *P* = 0.02 | *P* < 0.001 | *P* < 0.001 |
|  | 12 months | *P* = 0.50 | *P* = 0.38 | *P* = 0.47 |
| p-tau/tau | 6 months | *P* = 0.01 | *P* < 0.001 | *P* < 0.001 |
|  | 12 months | *P* = 0.02 | *P* = 0.07 | *P* = 0.20 |

1-4. Positive Nissl cells, GFAP and Iba1 expression levels in the cortex and hippocampus

| Parameters | Intervention period | Cortex | | | | Hippocampal | | | |
| --- | --- | --- | --- | --- | --- | --- | --- | --- | --- |
|  |  | Factors | | Interactions | | Factors | | Interactions | |
|  |  | genotype | diet | | genotype🞨diet | genotype | diet | | genotype🞨diet |
| Positive Nissl cell number | 6 months | *P <* 0.001 | *P* = 0.35 | | *P* = 0.12 | *P* = 0.002 | *P <* 0.001 | | *P* = 0.16 |
|  | 12 months | *P <* 0.001 | *P* = 0.16 | | *P* = 0.94 | *P <* 0.001 | *P* = 0.03 | | *P* = 0.63 |
| GFAP expression level | 6 months | *P* = 0.07 | *P* = 0.04 | | *P* = 0.28 | *P <* 0.001 | *P* = 0.007 | | *P* = 0.02 |
|  | 12 months | *P <* 0.001 | *P* = 0.002 | | *P* = 0.001 | *P* = 0.02 | *P* = 0.42 | | *P* = 0.07 |
| Iba1 expression level | 6 months | *P* = 0.83 | *P* = 0.006 | | *P* = 0.01 | *P* = 0.05 | *P* = 0.79 | | *P* = 0.02 |
|  | 12 months | *P* = 0.10 | *P* = 0.11 | | *P* = 0.002 | *P* = 0.50 | *P* = 0.15 | | *P* = 0.12 |

1-5. *Lpin1, Dgat1, Atgl, nSMase2, Cers3, Acer3, Sgms2, Gal3st1, Arsa, Psap mRNA expression level*

| Parameters | Intervention period | Factors | | Interactions |
| --- | --- | --- | --- | --- |
|  |  | genotype | diet | genotype🞨diet |
| *Lpin1* | 6 months | *P* = 0.50 | *P* = 0.32 | *P* = 0.14 |
|  | 12 months | *P* < 0.001 | *P* < 0.001 | *P* < 0.001 |
| *Dgat1* | 6 months | *P* = 0.20 | *P* = 0.35 | *P* < 0.001 |
|  | 12 months | *P* < 0.001 | *P* < 0.001 | *P* = 0.49 |
| *Atgl* | 6 months | *P* < 0.001 | *P* < 0.001 | *P* < 0.001 |
|  | 12 months | *P* = 0.09 | *P* < 0.001 | *P* = 0.10 |
| *nSMase2* | 6 months | *P* < 0.001 | *P* < 0.001 | *P* < 0.001 |
|  | 12 months | *P* < 0.001 | *P* < 0.001 | *P* = 0.03 |
| *Cers3* | 6 months | *P* = 0.11 | *P* = 0.31 | *P* = 0.13 |
|  | 12 months | *P* = 0.48 | *P >* 0.99 | *P* = 0.44 |
| *Acer3* | 6 months | *P* < 0.001 | *P* = 0.02 | *P* < 0.001 |
|  | 12 months | *P* = 0.06 | *P* < 0.001 | *P* = 0.23 |
| *Sgms2* | 6 months | *P* < 0.001 | *P =* 0.52 | *P =* 0.005 |
|  | 12 months | *P* = 0.004 | *P* < 0.001 | *P* = 0.21 |
| *Gal3st* | 6 months | *P* < 0.001 | *P* < 0.001 | *P* < 0.001 |
|  | 12 months | *P* < 0.001 | *P* < 0.001 | *P* < 0.001 |
| *Arsa* | 6 months | *P* < 0.001 | *P* < 0.001 | *P* < 0.001 |
|  | 12 months | *P* = 0.003 | *P* < 0.001 | *P* < 0.001 |
| *Psap* | 6 months | *P* < 0.001 | *P* < 0.001 | *P* < 0.001 |
|  | 12 months | *P* < 0.001 | *P* < 0.001 | *P* = 0.002 |

ApoE: Apolipoprotein E; Aβ: Amyloid-beta Protein; APP: Amyloid Precursor Protein; BACE1: Beta-Site Amyloid Precursor Protein Cleaving Enzyme 1; LRP1: Low-Density Lipoprotein Receptor-Related Protein 1; IDE: Insulin-Degrading Enzyme; TAU: Tau Protein; GFAP: Glial Fibrillary Acidic Protein; Iba1: Ionized Calcium - Binding Adapter Molecule 1. nSMase2: neutral Sphingomyelinase 2; Cers3: Ceramide Synthase 3; Acer3: Alkaline Ceramidase 3; Sgms2: Sphingomyelin Synthase 2; Lpin1: Lipin 1; Dgat1: Diacylglycerol Acyltransferase 1; Atgl: Adipose Triglyceride Lipase; Gal3st1: Galactose-3-O-Sulfotransferase 1; Arsa: Arylsulfatase A; Psap: Prosaposin

**Supplementary Table 2**. **Contents of various lipids in each group (%)**

| Lipids | E3-CON | E3-DHA | E4-CON | E4-DHA | E3-CON-N | E3-DHA-N | E4-CON-N | E4-DHA-N | *P-value* |
| --- | --- | --- | --- | --- | --- | --- | --- | --- | --- |
| DHA | 0.0367±0.0016 | 0.0459±0.0019_a_ | 0.0355±0.0012 | 0.0456±0.0009_c_ | 0.0088±0.0009_a_ | 0.0078±0.0011_b_ | 0.0075±0.0006_c,e_ | 0.0072±0.0012_d_ | <0.0001 |
| AA | 0.0614±0.026 | 0.0476±0.0035_a_ | 0.0583±0.0021 | 0.0442±0.0013_c_ | 0.0123±0.0017_a_ | 0.0083±0.0019_b,e_ | 0.0066±0.0005_c_ | 0.0056±0.0005_d_ | <0.0001 |
| PA | 0.0576±0.0027 | 0.0577±0.0038 | 0.0496±0.0021 | 0.0561±0.0044 | 2.633±0.9964_a_ | 3.294±1.245_b_ | 5.738±0.4135_c,e_ | 5.069±0.968_d,f_ | <0.0001 |
| n-3 PUFA | 0.0391±0.017 | 0.0531±0.0024_a_ | 0.0376±0.0011 | 0.0527±0.0016_c_ | 0.0199±0.0026_a_ | 0.0271±0.0096_b_ | 0.0258±0.0018_c_ | 0.0230±0.0028_d_ | <0.0001 |
| n-6 PUFA | 0.0689±0.0031 | 0.0541±0.0038 | 0.0650±0.0023 | 0.0497±0.0013 | 0.1087±0.0217 | 0.1470±0.0517_b_ | 0.2085±0.0111_c,e_ | 0.1946±0.0354_d_ | 0.0001 |
| SFA | 0.0842±0.0038 | 0.0815±0.0047 | 0.0717±0.0038 | 0.0777±0.0060 | 3.244±1.366 | 4.428±1.732_b_ | 6.830±0.7934_c,e_ | 6.041±1.271_d_ | <0.0001 |
| SA | 0.0252±0.0016 | 0.0227±0.0012 | 0.0210±0.0019 | 0.0207±0.0024 | 0.5524±0.4167 | 1.025±0.4746_b_ | 0.9524±0.4017_c_ | 0.8796±0.2900_d_ | 0.0339 |
| PC | 0.9937±0.0002 | 0.9942±0.0005 | 0.9930±0.0002 | 0.9938±0.0002 | 0.9957±0.0004_a_ | 0.9960±0.0006_b_ | 0.9966±0.0001_c_ | 0.9962±0.0006_d_ | <0.0001 |
| PE | 0.3815±0.0073 | 0.4171±0.0201 | 0.3949±0.0228 | 0.3764±0.0080 | 0.3344±0.0227 | 0.2404±0.0578_b_ | 0.2092±0.0415_c,e_ | 0.2585±0.0641_d_ | 0.0019 |
| PS | 0.1120±0.0139 | 0.1154±0.0100 | 0.1048±0.0124 | 0.1304±0.0108 | 0.4410±0.0063_a_ | 0.4615±0.0248_b_ | 0.5284±0.0581_c,e_ | 0.5171±0.0410_d_ | <0.0001 |
| PI | 0.0731±0.0050 | 0.0805±0.0065 | 0.0750±0.0053 | 0.0771±0.0039 | 0.1153±0.0016_a_ | 0.1238±0.0073_b_ | 0.1240±0.0042_c_ | 0.1323±0.0117_d_ | <0.0001 |
| PG | 0.0011±0.0001 | 0.0011±0.0001 | 0.0012±0.0001 | 0.0009±0.0001 | 0.0009±0.0001_a_ | 0.0007±0.0001_b_ | 0.0005±0.0001_c,e_ | 0.0005±0.0001_d_ | <0.0001 |
| CL | 0.0099±0.0012 | 0.0099±0.0007 | 0.0091±0.0012 | 0.0111±0.0007 | 0.0390±0.0054_a_ | 0.0328±0.0083_b_ | 0.0229±0.0037_c,e_ | 0.0306±0.0081_d_ | 0.0002 |
| Chol | 3.381±0.1758 | 3.408±0.1517 | 3.390±0.2187 | 3.605±0.2298 | 2.097±0.4339_a_ | 1.288±0.6374_b,e_ | 0.5674±0.0990_c,e_ | 0.7438±0.2440_d_ | <0.0001 |
| SM | 0.0705±0.0071 | 0.0753±0.0062 | 0.0864±0.0080 | 0.0716±0.0051 | 0.0864±0.0209 | 0.0805±0.0225 | 0.0563±0.0037 | 0.0702±0.0194 | 0.8083 |
| Cer | 0.1169±0.0116 | 0.1766±0.0382 | 0.1532±0.0363 | 0.1255±0.0228 | 0.1337±0.0445 | 0.1000±0.0564 | 0.0239±0.0075 | 0.0665±0.0339 | 0.1091 |
| GluCer | 0.0002±0.0001 | 0.0003±0.0001 | 0.0003±0.0001 | 0.0002±0.0001 | 0.0015±0.0001_a_ | 0.0018±0.0001_b_ | 0.0028±0.0005_c,e_ | 0.0021±0.0001_d,g_ | <0.0001 |
| GalCer | 0.0098±0.0012 | 0.0115±0.0014 | 0.0131±0.0010 | 0.0104±0.0002 | 0.0079±0.0017 | 0.0121±0.0045 | 0.0061±0.0012 | 0.0082±0.0025 | 0.2993 |
| LacCer | 0.0010±0.0001 | 0.0011±0.0001 | 0.0012±0.0001 | 0.0011±0.0001 | 0.0015±0.0003 | 0.0016±0.0003 | 0.0014±0.0002 | 0.0016±0.0003 | 0.3073 |

Data were expressed as mean ± SEM. E3-CON: ApoE3 control mice, lipid raft fraction; E3-DHA: ApoE3 mice, DHA treatment, lipid raft fraction; E4-CON: ApoE4 control mice, lipid raft fraction; E4-DHA: ApoE4 mice, DHA treatment, lipid raft fraction; E3-CON-N: ApoE3 control mice, non-lipid raft fraction; E3-DHA-N: ApoE3 mice, DHA treatment, non-lipid raft fraction; E4-CON-N: ApoE4 control mice, non-lipid raft fraction; E4-DHA-N: ApoE4 mice, DHA treatment, non-lipid raft fraction. PA, phosphatidic acid; SA, stearic acid; FFA, free fatty acid; AA, arachidonic acid; DHA, docosahexaenoic acid; PUFA, polyunsaturated fatty acid; PC, phosphatidylcholine; PE, phosphatidylethanolamine; PS, phosphatidylserine; PI, phosphatidylinositol; PG, phosphatidylglycerol; Chol, cholesterol; GP, glycophospholipid; Cer, ceramide; SM, sphingomyelin; GSL, glycosphingolipid. a, *P* < 0.05 vs. E3-CON group; b, *P* < 0.05 vs. E3-DHA group; c, *P* < 0.05 vs. E4-CON group; d, *P* < 0.05 vs. E4-DHA group; e, *P* < 0.05 vs. E3-CON-N group; f, *P* < 0.05 vs. E3-DHA-N group; g, *P* < 0.05 vs. E4-CON-N group.

**Supplementary Table 3. Values shown in Fig.4**

| Class | ApoE4-CON vs. ApoE3-CON | ApoE4-CON-N vs. ApoE3-CON-N | ApoE3-CON-N vs. ApoE3-CON | ApoE4-CON-N vs. ApoE4-CON |
| --- | --- | --- | --- | --- |
| CL | 0 | 11 | 65 | 24 |
| TAG | 1 | 10 | 57 | 66 |
| DAG | 0 | 9 | 24 | 28 |
| PC | 2 | 24 | 23 | 31 |
| PS | 0 | 1 | 17 | 17 |
| GluCer | 0 | 2 | 14 | 14 |
| PI | 0 | 0 | 13 | 12 |
| CE | 1 | 3 | 12 | 13 |
| PI-FA | 0 | 0 | 11 | 16 |
| SM | 0 | 1 | 11 | 9 |
| PC-O | 1 | 2 | 9 | 11 |
| Cer | 0 | 8 | 9 | 17 |
| PA | 0 | 0 | 9 | 10 |
| FFA | 0 | 4 | 8 | 11 |
| PC-FA | 0 | 0 | 8 | 7 |
| SL | 7 | 0 | 7 | 5 |
| GM3 | 0 | 0 | 7 | 7 |
| PS-FA | 0 | 0 | 7 | 7 |
| PA-FA | 0 | 0 | 7 | 6 |
| PE-O-FA | 1 | 5 | 6 | 18 |
| GalCer | 0 | 5 | 6 | 18 |
| PE-FA | 0 | 14 | 5 | 25 |
| PE | 0 | 5 | 5 | 9 |
| PC-O-FA | 0 | 0 | 5 | 6 |
| LacCer | 0 | 2 | 5 | 5 |
| BMP | 0 | 0 | 4 | 5 |
| AC | 0 | 1 | 3 | 7 |
| LPI | 0 | 3 | 3 | 4 |
| LPE | 0 | 1 | 3 | 3 |
| LPS | 0 | 1 | 3 | 3 |
| Sph | 0 | 0 | 3 | 3 |
| PE-O | 0 | 3 | 2 | 15 |
| LPA | 0 | 1 | 2 | 2 |
| LPC | 0 | 3 | 1 | 5 |
| PG | 0 | 4 | 0 | 10 |
| Free Cho | 0 | 1 | 0 | 1 |

| Class | ApoE3-DHA vs. ApoE3-CON | ApoE4-DHA vs. ApoE4-CON | ApoE4-DHA-N vs. ApoE4-CON-N | ApoE3-DHA-N vs. ApoE3-CON-N |
| --- | --- | --- | --- | --- |
| PG | 4 | 9 | 1 | 0 |
| BMP | 6 | 6 | 0 | 2 |
| SL | 0 | 0 | 0 | 0 |
| CL | 8 | 13 | 0 | 0 |
| FFA | 3 | 6 | 0 | 0 |
| PE-O-FA | 7 | 13 | 0 | 1 |
| PE-FA | 9 | 22 | 1 | 6 |
| PE-O | 8 | 11 | 0 | 0 |
| PE | 8 | 8 | 1 | 3 |
| PI-FA | 8 | 9 | 2 | 7 |
| PI | 5 | 2 | 1 | 4 |
| GM3 | 3 | 0 | 0 | 2 |
| PC-O-FA | 5 | 5 | 1 | 3 |
| PC-FA | 21 | 22 | 6 | 7 |
| LPI | 0 | 0 | 0 | 0 |
| LPE | 1 | 1 | 0 | 0 |
| LPA | 0 | 0 | 0 | 0 |
| PA-FA | 0 | 0 | 0 | 0 |
| PA | 0 | 0 | 0 | 0 |
| LPS | 0 | 0 | 0 | 0 |
| PS-FA | 2 | 3 | 2 | 4 |
| PS | 2 | 4 | 5 | 5 |
| Sph | 0 | 1 | 0 | 0 |
| Cer | 1 | 0 | 0 | 0 |
| GluCer | 0 | 6 | 0 | 0 |
| GalCer | 0 | 6 | 0 | 1 |
| LacCer | 1 | 0 | 0 | 0 |
| AC | 0 | 1 | 0 | 0 |
| LPC | 1 | 1 | 0 | 0 |
| PC-O | 4 | 6 | 0 | 0 |
| PC | 11 | 15 | 0 | 1 |
| SM | 7 | 0 | 0 | 0 |
| DAG | 4 | 12 | 2 | 1 |
| TAG | 11 | 22 | 1 | 2 |
| CE | 10 | 8 | 1 | 0 |
| Free Cho | 0 | 0 | 0 | 0 |

| Class | E4-DHA vs. E3-DHA | E4-DHA-N vs. E3-DHA-N | E3-DHA-N vs. E3-DHA | E4-DHA-N vs. E4-DHA |
| --- | --- | --- | --- | --- |
| TAG | 33 | 0 | 55 | 68 |
| CL | 0 | 0 | 26 | 23 |
| PC | 1 | 0 | 21 | 29 |
| DAG | 2 | 1 | 19 | 25 |
| PS | 0 | 1 | 17 | 17 |
| PI-FA | 2 | 0 | 15 | 16 |
| GluCer | 0 | 0 | 13 | 14 |
| FFA | 0 | 0 | 10 | 11 |
| PA | 0 | 0 | 10 | 10 |
| PI | 1 | 0 | 9 | 14 |
| PC-FA | 13 | 0 | 9 | 5 |
| GalCer | 0 | 0 | 9 | 13 |
| PC-O | 2 | 0 | 8 | 7 |
| CE | 2 | 0 | 8 | 9 |
| PE | 1 | 0 | 7 | 6 |
| PC-O-FA | 2 | 1 | 7 | 6 |
| PA-FA | 0 | 1 | 7 | 7 |
| PS-FA | 0 | 0 | 7 | 6 |
| PG | 5 | 0 | 6 | 8 |
| GM3 | 0 | 1 | 6 | 7 |
| Cer | 0 | 0 | 6 | 11 |
| PE-O-FA | 1 | 0 | 5 | 12 |
| LacCer | 1 | 0 | 5 | 7 |
| SM | 5 | 0 | 5 | 11 |
| PE-FA | 0 | 0 | 4 | 14 |
| LPI | 1 | 0 | 4 | 4 |
| BMP | 1 | 0 | 3 | 5 |
| SL | 0 | 0 | 3 | 4 |
| PE-O | 1 | 0 | 3 | 7 |
| LPS | 0 | 0 | 3 | 3 |
| LPA | 0 | 0 | 2 | 2 |
| Sph | 0 | 0 | 2 | 4 |
| LPC | 1 | 0 | 2 | 3 |
| LPE | 0 | 0 | 1 | 2 |
| AC | 0 | 0 | 1 | 2 |
| Free Cho | 2 | 0 | 1 | 1 |

**Supplementary Table 4. Comparison of Differential Lipids in Fig.4**

| Lipid name | E4CN | E3CN | *P-value* | Fold change | Down/Up |
| --- | --- | --- | --- | --- | --- |
| Cer d18:1/24:1 | 0.0015±0.0016 | 0.017±0.023 | 0.028571 | 0.088591 | down |
| Cer d18:0/24:1 | 0.000052±0.000035 | 0.00055±0.00073 | 0.028571 | 0.095008 | down |
| Cer d20:1/18:0 | 0.0015±0.0018 | 0.012±0.014 | 0.028571 | 0.125835 | down |
| Cer d18:1/20:0 | 0.00072±0.00081 | 0.0049±0.0063 | 0.028571 | 0.147341 | down |
| Cer d18:1/22:0 | 0.00072±0.00091 | 0.0045±0.0062 | 0.028571 | 0.157979 | down |
| Cer d17:0/22:0 | 0.000061±0.000063 | 0.00037±0.00045 | 0.028571 | 0.161946 | down |
| Cer d18:0/18:0 | 0.000058±0.000028 | 0.000043±0.000042 | 0.028571 | 0.167881 | down |
| Cer d18:1/18:0 | 0.00029±0.00019 | 0.00054±0.00052 | 0.028571 | 0.185068 | down |
| DAG38:6(16:0/22:6) | 0.000078±0.000079 | 0.00033±0.00020 | 0.028571 | 0.236907 | down |
| DAG38:4(18:0/20:4) | 0.00033±0.00035 | 0.0014±0.00087 | 0.028571 | 0.244847 | down |
| PC40:0 | 0.0010±0.00080 | 0.00017±0.00018 | 0.028571429 | 5.840258971 | Up |
| CE-18:3 | 0.0020±0.00089 | 0.00063±0.00042 | 0.028571429 | 3.121654887 | Up |
| CE-16:0 | 0.015±0.0051 | 0.0050±0.0032 | 0.028571429 | 3.004921437 | Up |
| CE-16:1 | 0.027±0.013 | 0.010±0.0083 | 0.028571429 | 2.712509304 | Up |
| TAG56:3(20:1) | 0.00011±0.000076 | 0.000041±0.000026 | 0.028571429 | 2.671620456 | Up |
| GluCer d18:1/24:0 | 0.00038±0.00022 | 0.00014±0.000088 | 0.028571429 | 2.623543978 | Up |
| TAG52:2(18:2) | 0.00031±0.00011 | 0.00012±0.000094 | 0.028571429 | 2.555813016 | Up |
| LPS18:0 | 0.0057±0.0028 | 0.0022±0.0018 | 0.028571429 | 2.550548728 | Up |
| LPI20:4 | 0.00056±0.00025 | 0.00022±0.00014 | 0.028571429 | 2.543158409 | Up |
| TAG54:6(18:2) | 0.0018±0.00051 | 0.00073±0.00071 | 0.028571429 | 2.520185952 | Up |

| Lipid name | E4C | E3C | *P-value* | Fold change | Down/Up |
| --- | --- | --- | --- | --- | --- |
| PC32:1 | 0.010±0.00063 | 0.011±0.00091 | 0.028571 | 0.905695 | down |
| PC34:3 | 0.00033±0.000022 | 0.00037±0.000016 | 0.028571 | 0.908802 | down |
| SL d18:1/20:0 | 0.00027±0.000059 | 0.00019±0.000012 | 0.028571 | 1.455397 | Up |
| SL d18:1/22:0 | 0.0011±0.00023 | 0.00080±0.00014 | 0.028571 | 1.392856 | Up |
| SL d18:1/22:1 | 0.00047±0.000073 | 0.00035±0.000035 | 0.028571 | 1.353447 | Up |
| SL d18:1/24:0 | 0.0046±0.00082 | 0.0035±0.00018 | 0.028571 | 1.318487 | Up |
| CE-20:3 | 0.00031±0.000051 | 0.00023±0.000034 | 0.028571 | 1.309666 | Up |
| SL d18:1/20:1 | 0.000024±0.0000023 | 0.000019±0.0000027 | 0.028571 | 1.278447 | Up |
| SL d18:1/24:1 | 0.012±0.0018 | 0.0095±0.00089 | 0.028571 | 1.27511 | Up |
| SL d18:1/24:1h | 0.0038±0.00058 | 0.0030±0.00033 | 0.028571 | 1.262542 | Up |
| PE O-40:4(O-20:1_20:3) | 0.000022±0.0000024 | 0.000019±0.0000026 | 0.028571 | 1.171958 | Up |
| PC O-40:4 | 0.000090±0.0000072 | 0.000077±0.000020 | 0.028571 | 1.169964 | Up |

| Lipid name | E4CN | E4C | *P-value* | Fold change | Down/Up |
| --- | --- | --- | --- | --- | --- |
| Cer d18:0/24:1 | 0.000052±0.000035 | 0.0036±0.0023 | 0.028571 | 0.014499 | down |
| Cer d20:0/22:1 | 0.000025±0.0000086 | 0.0013±0.00056 | 0.028571 | 0.019622 | down |
| Cer d18:1/24:1 | 0.0013±0.0010 | 0.000033±0.0000087 | 0.028571 | 0.021596 | down |
| Cer d18:0/26:0 | 0.000061±0.000092 | 0.0027±0.0017 | 0.028571 | 0.022366 | down |
| Cer d17:0/24:1 | 0.000058±0.000061 | 0.0024±0.0014 | 0.028571 | 0.024394 | down |
| Cer d20:1/24:1 | 0.000071±0.000079 | 0.0012±0.0011 | 0.028571 | 0.058171 | down |
| Cer d18:1/25:1 | 0.000095±0.00011 | 0.0014±0.0011 | 0.028571 | 0.066192 | down |
| DAG38:6(16:0/22:6) | 0.000078±0.000079 | 0.00089±0.00026 | 0.028571 | 0.087704 | down |
| DAG38:4(18:0/20:4) | 0.00033±0.00035 | 0.0036±0.00062 | 0.028571 | 0.091893 | down |
| BMP38:5(18:1_20:4) | 0.0000042±0.0000019 | 0.000040±0.0000075 | 0.028571 | 0.104978 | down |
| FFA14:0 | 0.14±0.022 | 0.0012±0.00060 | 0.028571 | 120.1102 | Up |
| FFA16:0 | 5.74±1.32 | 0.050±0.0066 | 0.028571 | 115.7671 | Up |
| TAG54:6(18:2) | 0.0018±0.00051 | 0.000016±0.0000042 | 0.028571 | 113.996 | Up |
| LPA16:0 | 0.00038±0.00011 | 0.0000043±0.0000022 | 0.028571 | 87.76406 | Up |
| TAG54:0(18:0) | 0.00084±0.00027 | 0.0000097±0.0000040 | 0.028571 | 86.89843 | Up |
| LPA18:0 | 0.00093±0.00018 | 0.000014±0.0000043 | 0.028571 | 68.70252 | Up |
| TAG46:1(14:0) | 0.00041±0.00013 | 0.0000072±0.0000048 | 0.028571 | 57.28206 | Up |
| TAG46:1(16:1) | 0.00055±0.00015 | 0.0000099±0.0000071 | 0.028571 | 55.80127 | Up |
| PC36:5 | 0.056±0.0053 | 0.0010±0.00016 | 0.028571 | 54.82355 | Up |
| DAG36:4(18:2/18:2) | 0.00040±0.000089 | 0.0000073±0.0000028 | 0.028571 | 54.60748 | Up |

| Lipid name | E3CN | E3C | *P-value* | Fold change | Down/Up |
| --- | --- | --- | --- | --- | --- |
| BMP38:6(18:2_20:4) | 0.0000017±0.0000015 | 0.0000066±0.0000019 | 0.028571 | 0.257057 | down |
| BMP36:3(18:2_18:1) | 0.0000084±0.0000068 | 0.000035±0.0000073 | 0.028571 | 0.243579 | down |
| FFA22:6 | 0.0088±0.0027 | 0.037±0.0052 | 0.028571 | 0.24107 | down |
| BMP38:5(18:1_20:4) | 0.0000076±0.0000031 | 0.000034±0.0000089 | 0.028571 | 0.223026 | down |
| FFA20:4 | 0.012±0.0053 | 0.061±0.0082 | 0.028571 | 0.19988 | down |
| Cer d18:0/24:1 | 0.00055±0.00073 | 0.0032±0.0014 | 0.028571 | 0.172058 | down |
| Cer d17:0/24:1 | 0.00020±0.00026 | 0.0025±0.0016 | 0.028571 | 0.078748 | down |
| Cer d18:0/26:0 | 0.00019±0.00025 | 0.0029±0.0022 | 0.028571 | 0.065905 | down |
| Cer d20:0/22:1 | 0.000061±0.00011 | 0.0012±0.00068 | 0.028571 | 0.052139 | down |
| PE36:4 | 0.00±0.00 | 0.0078±0.00087 | 0.021071 | 0 | down |
| GluCer d18:0/20:0 | 0.000040±0.000063 | 0.00000077±0.00000034 | 0.028571 | 52.77596 | Up |
| FFA16:0 | 2.63±3.17 | 0.058±0.0086 | 0.028571 | 45.6969 | Up |
| FFA14:0 | 0.059±0.056 | 0.0013±0.00032 | 0.028571 | 44.47794 | Up |
| LPA16:0 | 0.00018±0.00017 | 0.0000041±0.0000018 | 0.028571 | 43.47913 | Up |
| TAG54:0(18:0) | 0.00036±0.00034 | 0.000010±0.0000024 | 0.028571 | 35.36191 | Up |
| TAG54:6(18:2) | 0.00073±0.00071 | 0.000023±0.0000083 | 0.028571 | 31.66322 | Up |
| FFA16:1 | 0.0017±0.0032 | 0.0025±0.00077 | 0.028571 | 27.91214 | Up |
| LPA18:0 | 0.00038±0.00035 | 0.000014±0.0000037 | 0.028571 | 27.6819 | Up |
| TAG46:1(16:1) | 0.00038±0.00040 | 0.000014±0.0000021 | 0.028571 | 26.90391 | Up |
| PC36:5 | 0.028±0.027 | 0.0011±0.00016 | 0.028571 | 24.69498 | Up |

| Lipid name | E4DN | E3DN | *P-value* | Fold change | Down/Up |
| --- | --- | --- | --- | --- | --- |
| PA 38:5(18:1_20:4) | 0.00054±0.00018 | 0.00083±0.00028 | 0.028571 | 0.650676 | down |
| PC O-34:2(O-16:1_18:1) | 0.00020±0.000083 | 0.00034±0.000067 | 0.028571 | 0.605275 | down |
| NeuAcHex2Cer 40:1;O2 | 0.00041±0.00015 | 0.00083±0.00055 | 0.028571 | 0.493025 | down |
| DAG36:4(16:0/20:4) | 0.000022±0.000015 | 0.000066±0.000036 | 0.028571 | 0.331071 | down |
| PS 32:0 | 0.00010±0.000061 | 0.000047±0.000023 | 0.028571 | 2.137631 | Up |

| Lipid name | E4D | E3D | *P-value* | Fold change | Down/Up |
| --- | --- | --- | --- | --- | --- |
| TAG52:4(18:2) | 0.000025±0.000014 | 0.000056±0.000029 | 0.028571 | 0.450893 | down |
| TAG52:3(18:2) | 0.000035±0.000017 | 0.000079±0.000048 | 0.028571 | 0.44607 | down |
| SM 34:2;O2 | 0.000046±0.000024 | 0.00011±0.00012 | 0.028571 | 0.400871 | down |
| TAG52:3(16:0) | 0.000042±0.000028 | 0.00011±0.000065 | 0.028571 | 0.398157 | down |
| SM 38:2;O2 | 0.00032±0.00027 | 0.00086±0.00077 | 0.028571 | 0.37772 | down |
| TAG54:4(18:2) | 0.000038±0.000021 | 0.00010±0.000053 | 0.028571 | 0.371026 | down |
| TAG54:4(18:1) | 0.000075±0.000042 | 0.00021±0.000096 | 0.028571 | 0.361488 | down |
| TAG52:4(18:1) | 0.000016±0.000010 | 0.000045±0.000023 | 0.028571 | 0.357501 | down |
| TAG52:4(16:1) | 0.000014±0.000010 | 0.000040±0.000020 | 0.028571 | 0.347398 | down |
| SM 40:2;O2 | 0.00054±0.00056 | 0.0016±0.0016 | 0.028571 | 0.33497 | down |
| PC O-40:7 | 0.00026±0.00012 | 0.00018±0.0000080 | 0.028571 | 1.41137 | UP |
| PC O-40:6 | 0.00027±0.000073 | 0.00022±0.000010 | 0.028571 | 1.245765 | UP |

| Lipid name | E4DN | E4D | *P-value* | Fold change | Down/Up |
| --- | --- | --- | --- | --- | --- |
| DAG36:4(16:0/20:4) | 0.000022±0.000015 | 0.00015±0.000025 | 0.028571 | 0.145948 | down |
| DAG38:4(18:0/20:4) | 0.00038±0.00047 | 0.0027±0.00044 | 0.028571 | 0.142761 | down |
| FFA20:4 | 0.0056±0.0016 | 0.044±0.0042 | 0.028571 | 0.127195 | down |
| Cer d18:1/24:1 | 0.0061±0.012 | 0.054±0.037 | 0.028571 | 0.113327 | down |
| Cer d20:1/24:1 | 0.00012±0.00017 | 0.0011±0.00093 | 0.028571 | 0.10567 | down |
| DAG38:6(16:0/22:6) | 0.00013±0.00016 | 0.0013±0.00020 | 0.028571 | 0.104026 | down |
| Cer d18:0/24:1 | 0.00019±0.00029 | 0.0035±0.0022 | 0.028571 | 0.054734 | down |
| Cer d18:0/26:0 | 0.00012±0.000064 | 0.0029±0.0019 | 0.028571 | 0.040691 | down |
| Cer d17:0/24:1 | 0.000065±0.000085 | 0.0029±0.0019 | 0.028571 | 0.022746 | down |
| Cer d20:0/22:1 | 0.000025±0.000018 | 0.0013±0.00090 | 0.028571 | 0.019039 | down |
| FFA14:0 | 0.093±0.048 | 0.00090±0.00035 | 0.028571 | 103.4586 | Up |
| TAG54:6(18:2) | 0.0014±0.00069 | 0.000015±0.0000033 | 0.028571 | 94.76176 | Up |
| TAG54:0(18:0) | 0.00076±0.00039 | 0.0000080±0.0000018 | 0.028571 | 94.10386 | Up |
| FFA16:0 | 5.06±3.08 | 0.056±0.014 | 0.028571 | 90.38515 | Up |
| LPA16:0 | 0.00036±0.00022 | 0.0000047±0.0000010 | 0.028571 | 76.98737 | Up |
| DAG36:4(18:2/18:2) | 0.00032±0.00018 | 0.0000052±0.00000087 | 0.028571 | 60.68249 | Up |
| LPA18:0 | 0.00080±0.00035 | 0.000013±0.0000042 | 0.028571 | 60.575 | Up |
| TAG54:5(18:1) | 0.0010±0.00040 | 0.000017±0.0000084 | 0.028571 | 59.19522 | Up |
| TAG52:4(16:0) | 0.00052±0.00043 | 0.0000093±0.0000043 | 0.029401 | 55.82509 | Up |
| FFA16:1 | 0.11±0.060 | 0.0019±0.00036 | 0.028571 | 55.3963 | Up |

| Lipid name | E3DN | E3D | P-value | Fold change | Down/Up |
| --- | --- | --- | --- | --- | --- |
| Cer d20:0/22:1 | 0.000044±0.000045 | 0.0017±0.00076 | 0.028571 | 0.025492 | down |
| Cer d18:0/26:0 | 0.00013±0.00022 | 0.0040±0.00085 | 0.028571 | 0.033532 | down |
| Cer d17:0/24:1 | 0.00017±0.00032 | 0.0034±0.00052 | 0.028571 | 0.048269 | down |
| Cer d18:0/24:1 | 0.00047±0.00098 | 0.0052±0.0029 | 0.028571 | 0.089463 | down |
| Cer d20:1/24:1 | 0.00020±0.00031 | 0.0014±0.00055 | 0.028571 | 0.140313 | down |
| Cer d18:1/25:0 | 0.00021±0.00025 | 0.0015±0.00061 | 0.028571 | 0.140957 | down |
| 16:0-carnitine | 0.0000066±0.000012 | 0.000046±0.000013 | 0.029401 | 0.144975 | down |
| FFA22:6 | 0.0078±0.0036 | 0.046±0.0059 | 0.028571 | 0.169312 | down |
| DAG38:6(16:0/22:6) | 0.00023±0.00032 | 0.0013±0.00031 | 0.028571 | 0.169613 | down |
| FFA20:4 | 0.0083±0.0060 | 0.048±0.011 | 0.028571 | 0.174933 | down |
| FFA14:0 | 0.11±0.16 | 0.0011±0.00025 | 0.028571 | 96.43118 | Up |
| LPA16:0 | 0.00031±0.00035 | 0.0000044±0.0000013 | 0.028571 | 70.86397 | Up |
| TAG54:6(18:2) | 0.0011±0.0012 | 0.000016±0.0000046 | 0.028571 | 66.88942 | Up |
| TAG54:0(18:0) | 0.00059±0.00066 | 0.0000090±0.0000011 | 0.028571 | 66.06366 | Up |
| FFA16:0 | 3.29±3.96 | 0.058±0.012 | 0.028571 | 57.10662 | Up |
| FFA16:1 | 0.12±0.27 | 0.0024±0.00039 | 0.028571 | 52.84646 | Up |
| LPA18:0 | 0.00077±0.00088 | 0.000015±0.0000067 | 0.028571 | 50.91008 | Up |
| FFA18:0 | 1.02±1.51 | 0.023±0.0038 | 0.028571 | 45.13666 | Up |
| GluCer d18:0/20:0 | 0.000033±0.000043 | 0.00000091±0.00000036 | 0.028571 | 36.43836 | Up |
| CL74:11(18:2) | 0.0011±0.0012 | 0.000032±0.000010 | 0.028571 | 33.27108 | Up |

| Lipid name | E3DN | E3CN | P-value | Fold change | Down/Up |
| --- | --- | --- | --- | --- | --- |
| TAG54:4(20:4) | 0.000015±0.000017 | 0.000044±0.00001 | 0.028571 | 0.330494 | down |
| PE40:4(18:0_22:4) | 0.0017±0.0016 | 0.0046±0.00084 | 0.028571 | 0.374534 | down |
| BMP38:5(18:1_20:4) | 0.0000029±0.00000083 | 0.0000076±0.0000031 | 0.028571 | 0.380859 | down |
| PE40:4 | 0.0017±0.0016 | 0.0046±0.00084 | 0.028571 | 0.389724 | down |
| PE38:5(18:1_20:4) | 0.0050±0.0045 | 0.012±0.0028 | 0.028571 | 0.398812 | down |
| PE42:4(22:0_20:4) | 0.00018±0.00016 | 0.00044±0.00022 | 0.028571 | 0.403592 | down |
| PC36:5(20:4_16:1) | 0.00013±0.000049 | 0.00033±0.00013 | 0.028571 | 0.411653 | down |
| PS 38:5(18:1_20:4) | 0.000055±0.000035 | 0.00013±0.000026 | 0.028571 | 0.431172 | down |
| PE40:6(20:2_20:4) | 0.000034±0.000032 | 0.000079±0.0000051 | 0.028571 | 0.438714 | down |
| PC38:4 | 0.019±0.0046 | 0.031±0.0098 | 0.028571 | 0.448213 | down |
| PE O-36:6(O-16:1_20:5) | 0.000072±0.000045 | 0.000012±0.000017 | 0.028571 | 5.985291 | Up |
| PI38:5(18:0_20:5) | 0.0061±0.0011 | 0.0011±0.00090 | 0.028571 | 5.705617 | Up |
| PC36:5(20:5_16:0) | 0.0012±0.00039 | 0.00022±0.00019 | 0.028571 | 5.506787 | Up |
| TAG56:8(22:6) | 0.000041±0.000020 | 0.000018±0.000014 | 0.028571 | 2.21373 | Up |
| PS 38:3(18:0_20:3) | 0.000091±0.000026 | 0.000041±0.000047 | 0.028571 | 2.206005 | Up |
| NeuAcHex2Cer 38:1;O2 | 0.00075±0.00026 | 0.00035±0.00011 | 0.028571 | 2.134487 | Up |
| NeuAcHex2Cer 40:1;O2 | 0.00083±0.00055 | 0.00039±0.00023 | 0.028571 | 2.107165 | Up |
| PI34:2(18:2_16:0) | 0.00058±0.00023 | 0.00037±0.00012 | 0.028571 | 1.579398 | Up |
| PI36:1(18:1_18:0) | 0.010±0.0045 | 0.0070±0.0012 | 0.028571 | 1.482397 | Up |
| PI 38:6 | 0.0045±0.0013 | 0.0030±0.00055 | 0.028571 | 1.481778 | Up |

| Lipid name | E3D | E3C | P-value | Fold change | Down/Up |
| --- | --- | --- | --- | --- | --- |
| BMP38:6(18:2_20:4) | 0.0000011±0.00000045 | 0.0000066±0.0000019 | 0.028571 | 0.159849 | Down |
| TAG56:5(22:4) | 0.0000058±0.0000013 | 0.000016±0.0000045 | 0.028571 | 0.35679 | Down |
| PC42:7(20:4_22:3) | 0.000020±0.0000078 | 0.000056±0.000013 | 0.028571 | 0.364944 | Down |
| BMP36:3(18:2_18:1) | 0.000016±0.0000046 | 0.000035±0.0000073 | 0.028571 | 0.450938 | Down |
| PE40:4(18:0_22:4) | 0.0021±0.00076 | 0.0045±0.0013 | 0.028571 | 0.461786 | Down |
| BMP38:4(18:0_20:4) | 0.00000093±0.00000023 | 0.0000020±0.0000011 | 0.028571 | 0.46236 | Down |
| BMP38:5(18:1_20:4) | 0.000016±0.0000043 | 0.000034±0.0000089 | 0.028571 | 0.464912 | Down |
| FFA22:4 | 0.00042±0.00016 | 0.00088±0.00015 | 0.028571 | 0.480177 | Down |
| CE-22:4 | 0.00012±0.000039 | 0.00025±0.000071 | 0.028571 | 0.482426 | Down |
| PS 38:5(18:1_20:4) | 0.000044±0.0000096 | 0.000091±0.000020 | 0.028571 | 0.486453 | Down |
| PE O-36:6(O-16:1_20:5) | 0.00021±0.000084 | 0.0000089±0.0000023 | 0.028571 | 24.04939 | Up |
| CE-20:5 | 0.0029±0.0017 | 0.00018±0.000041 | 0.028571 | 16.11295 | Up |
| DAG38:5(18:0/20:5) | 0.00017±0.000052 | 0.000012±0.0000049 | 0.028571 | 13.92833 | Up |
| PI38:5(18:0_20:5) | 0.0037±0.00070 | 0.00038±0.00019 | 0.028571 | 9.788913 | Up |
| DAG38:6(18:1/20:5) | 0.000026±0.0000043 | 0.0000029±0.0000015 | 0.028571 | 8.697872 | Up |
| FFA20:5 | 0.0058±0.0014 | 0.00078±0.00014 | 0.028571 | 7.423252 | Up |
| PC36:5(20:5_16:0) | 0.0015±0.00017 | 0.00020±0.000044 | 0.028571 | 7.194657 | Up |
| NeuAcHex2Cer 40:2;O2 | 0.000082±0.000099 | 0.000014±0.000019 | 0.028571 | 5.914551 | Up |
| SM 40:2;O2 | 0.0016±0.0016 | 0.00035±0.00036 | 0.028571 | 4.525332 | Up |
| NeuAcHex2Cer 40:1;O2 | 0.00021±0.00023 | 0.000056±0.000041 | 0.028571 | 3.781347 | Up |

| Lipid name | E4DN | E4CN | P-value | Fold change | Down/Up |
| --- | --- | --- | --- | --- | --- |
| PC O-40:5(O-20:1_20:4) | 0.000021±0.000025 | 0.000093±0.000055 | 0.028571 | 0.230128 | down |
| PS 38:5(18:1_20:4) | 0.000055±0.000048 | 0.00021±0.00011 | 0.028571 | 0.264231 | down |
| PC38:6(20:4_18:2) | 0.000095±0.000071 | 0.00033±0.00013 | 0.028571 | 0.286652 | down |
| PC40:6(20:4_20:2) | 0.000058±0.000027 | 0.00017±0.000077 | 0.028571 | 0.347041 | down |
| PS 38:4(18:0_20:4) | 0.00034±0.000082 | 0.00071±0.00033 | 0.028571 | 0.475798 | down |
| PC38:5(20:4_18:1) | 0.0041±0.00098 | 0.0083±0.0024 | 0.028571 | 0.495275 | down |
| DAG34:2(16:1/18:1) | 0.00046±0.00031 | 0.00089±0.00012 | 0.028571 | 0.511382 | down |
| PE40:4(20:0_20:4) | 0.00013±0.000059 | 0.00024±0.000083 | 0.028571 | 0.524116 | down |
| CE-16:0 | 0.0080±0.0032 | 0.015±0.0051 | 0.028571 | 0.531188 | down |
| PS 40:4 | 0.0071±0.0017 | 0.013±0.0037 | 0.028571 | 0.537691 | down |
| PI38:5(18:0_20:5) | 0.0070±0.0029 | 0.0011±0.00024 | 0.028571 | 6.287956 | Up |
| PC36:5(20:5_16:0) | 0.0011±0.00035 | 0.00030±0.00020 | 0.028571 | 3.561532 | Up |
| PE34:2 | 0.00010±0.000071 | 0.000096±0.000081 | 0.028571 | 2.86428 | Up |
| PS 32:1 | 0.00022±0.000032 | 0.00012±0.000058 | 0.028571 | 1.839852 | Up |
| PG36:3(16:0_20:3) | 0.000016±0.0000055 | 0.0000092±0.0000044 | 0.042066 | 1.716843 | Up |
| PI34:2(18:1_16:1) | 0.00064±0.00013 | 0.00042±0.00013 | 0.028571 | 1.537571 | Up |
| PI 38:6 | 0.0043±0.0012 | 0.0031±0.00069 | 0.028571 | 1.385342 | Up |

| Lipid name | E4D | E4C | P-value | Fold change | Down/Up |
| --- | --- | --- | --- | --- | --- |
| BMP38:6(18:2_20:4) | 0.00000082±0.00000015 | 0.0000069±0.00000094 | 0.028571 | 0.119018 | down |
| TAG56:5(22:4) | 0.0000044±0.0000011 | 0.000020±0.0000044 | 0.028571 | 0.220228 | down |
| PC42:7(20:4_22:3) | 0.000015±0.0000089 | 0.000063±0.000021 | 0.028571 | 0.241653 | down |
| TAG56:6(22:5) | 0.000011±0.0000018 | 0.000040±0.0000071 | 0.028571 | 0.274766 | down |
| TAG56:6(20:4) | 0.0000079±0.0000015 | 0.000024±0.0000078 | 0.028571 | 0.322587 | down |
| BMP36:3(18:2_18:1) | 0.000011±0.0000017 | 0.000033±0.0000082 | 0.028571 | 0.330431 | down |
| CE-22:4 | 0.00013±0.000058 | 0.00036±0.000087 | 0.028571 | 0.352873 | down |
| PC40:6(20:4_20:2) | 0.000069±0.000012 | 0.00019±0.000043 | 0.028571 | 0.353669 | down |
| BMP38:5(18:1_20:4) | 0.000014±0.0000040 | 0.000040±0.0000075 | 0.028571 | 0.359168 | down |
| TAG54:5(20:4) | 0.000027±0.0000062 | 0.000076±0.000015 | 0.028571 | 0.363005 | down |
| PE O-36:6(O-16:1_20:5) | 0.00015±0.000050 | 0.0000092±0.0000025 | 0.028571 | 16.53377 | Up |
| CE-20:5 | 0.0025±0.0011 | 0.00018±0.000092 | 0.028571 | 13.80103 | Up |
| DAG38:5(18:0/20:5) | 0.00016±0.000048 | 0.000011±0.0000045 | 0.028571 | 13.75388 | Up |
| PI38:5(18:0_20:5) | 0.0044±0.0011 | 0.00036±0.000046 | 0.028571 | 12.12489 | Up |
| DAG38:6(18:1/20:5) | 0.000023±0.0000067 | 0.0000023±0.00000077 | 0.028571 | 10.02188 | Up |
| FFA20:5 | 0.0058±0.0024 | 0.00063±0.000098 | 0.028571 | 9.206236 | Up |
| PC36:5(20:5_16:0) | 0.0014±0.00036 | 0.00017±0.000037 | 0.028571 | 7.827717 | Up |
| PC36:5 | 0.0030±0.00088 | 0.0010±0.00016 | 0.028571 | 2.916739 | Up |
| CE-22:6 | 0.0094±0.0051 | 0.0036±0.0010 | 0.028571 | 2.629275 | Up |
| CL76:11(20:3) | 0.000073±0.000015 | 0.000028±0.0000064 | 0.028571 | 2.621743 | Up |

Data were expressed as mean ± SEM. E3C: ApoE3 control mice, lipid raft fraction; E3D: ApoE3 mice, DHA treatment, lipid raft fraction; E4C: ApoE4 control mice, lipid raft fraction; E4D: ApoE4 mice, DHA treatment, lipid raft fraction; E3CN: ApoE3 control mice, non-lipid raft fraction; E3DN: ApoE3 mice, DHA treatment, non-lipid raft fraction; E4CN: ApoE4 control mice, non-lipid raft fraction; E4DN: ApoE4 mice, DHA treatment, non-lipid raft fraction.

**Supplementary Table 5. Values corresponding to the heatmap in Fig.5**

|  |  | 6m | | | | | | | | | | | | | | | |
| --- | --- | --- | --- | --- | --- | --- | --- | --- | --- | --- | --- | --- | --- | --- | --- | --- | --- |
|  |  | ApoE3-CON | | | | ApoE3-DHA | | | | ApoE4-CON | | | | ApoE4-DHA | | | |
| TAG | MGLL | -0.646 | 0.420 | 0.034 | -2.008 | -0.448 | -0.313 | 1.242 | 0.912 | -0.102 | 0.559 | 2.452 | -0.220 | 0.524 | 0.552 | 0.783 | -0.208 |
|  | HSL | -0.985 | -0.634 | -0.490 | 1.540 | 0.020 | -0.002 | -0.646 | -1.924 | -0.636 | -0.165 | -2.132 | -0.200 | -0.908 | -1.599 | -0.401 | 0.496 |
|  | DGAT1 | 0.277 | -0.693 | -0.670 | 0.574 | 1.350 | -0.204 | 0.719 | 1.506 | 0.731 | 0.893 | -0.365 | -1.629 | 1.131 | 0.035 | -1.895 | -0.473 |
|  | GPAT1 | -0.250 | -2.902 | 0.021 | 0.848 | 0.515 | 1.011 | -0.282 | -0.578 | -0.879 | -0.485 | -2.709 | -0.357 | 0.177 | 0.286 | -0.428 | -0.085 |
| SL | PSAP | 0.114 | -0.643 | 0.882 | 0.883 | -1.401 | -0.266 | -1.302 | -0.457 | 0.379 | 0.568 | 0.055 | 1.401 | -1.702 | -0.665 | -1.954 | 2.961 |
|  | GALC | -0.277 | -0.583 | 1.099 | 0.769 | 0.800 | 0.751 | -0.141 | 0.226 | 0.285 | 0.719 | -0.985 | -1.524 | -0.068 | 0.591 | -0.602 | -2.134 |
|  | ARSA | -0.148 | -0.666 | 1.585 | 1.304 | -0.804 | -0.453 | -1.211 | -1.338 | 1.970 | 0.871 | -0.407 | 1.489 | -1.423 | -0.348 | -1.695 | 0.910 |
|  | CGT | -0.022 | -0.944 | 1.122 | 0.288 | 1.806 | 0.694 | 1.369 | -0.362 | 0.392 | -0.336 | 0.186 | -1.322 | 1.877 | -0.343 | -0.414 | -1.596 |
| CER | SPHK2 | 0.587 | -0.540 | -0.143 | -0.815 | 1.516 | 0.031 | 0.872 | 0.991 | 1.233 | 0.165 | 0.415 | -0.680 | 1.581 | 0.496 | 1.096 | -2.665 |
|  | SPHK1 | 1.362 | 0.637 | -0.267 | 0.407 | -0.178 | 0.410 | -1.456 | -0.485 | -0.866 | -0.657 | -0.721 | -0.003 | -0.010 | -1.306 | -0.505 | -0.556 |
|  | ASAH2 | -0.518 | 0.499 | 1.778 | -0.139 | 0.352 | 1.300 | 0.620 | -1.045 | 1.181 | -0.900 | -2.268 | -1.247 | 2.145 | 0.173 | -0.271 | -0.141 |
|  | ASAH1 | -0.771 | -0.286 | 2.352 | -0.133 | -1.171 | -0.573 | -0.494 | -1.103 | 0.215 | -0.316 | -0.261 | 0.695 | -1.632 | -0.217 | -1.990 | 2.442 |
|  | ASM | 0.064 | -0.564 | 1.868 | 1.065 | -1.115 | -0.312 | -1.321 | -0.942 | 0.909 | 0.204 | 0.030 | 1.245 | -1.535 | -0.570 | -2.345 | 2.544 |
|  | CERS2 | -0.508 | -0.650 | 0.923 | 1.285 | -0.410 | 0.596 | -1.107 | -2.439 | 0.992 | -0.194 | 0.063 | 0.318 | 0.125 | 0.152 | -1.962 | 2.157 |
|  | CERS1 | 0.709 | -0.269 | 2.139 | 0.689 | 0.260 | 0.664 | -0.218 | -0.917 | 0.978 | 0.074 | -0.482 | -0.275 | 0.185 | -0.266 | -1.170 | -1.016 |

|  |  | 12m | | | | | | | | | | | | | | | |
| --- | --- | --- | --- | --- | --- | --- | --- | --- | --- | --- | --- | --- | --- | --- | --- | --- | --- |
|  |  | ApoE3-CON | | | | ApoE3-DHA | | | | ApoE4-CON | | | | ApoE4-DHA | | | |
| TAG | MGLL | -1.859 | 0.195 | -0.335 | -2.081 | -1.140 | -1.502 | 0.838 | -0.547 | 1.233 | 0.096 | -0.495 | -0.130 | 1.009 | 1.113 | 0.556 | -0.483 |
|  | HSL | 1.961 | 0.334 | 0.276 | 0.330 | 1.479 | 0.519 | 0.256 | 2.007 | -0.057 | 0.477 | 0.408 | 1.249 | -0.219 | -1.309 | 0.144 | 0.809 |
|  | DGAT1 | 0.328 | 0.310 | -0.699 | -1.079 | -1.979 | 0.460 | -0.633 | -0.565 | 0.344 | 1.478 | -0.482 | -1.560 | -0.727 | 1.699 | 1.089 | 0.724 |
|  | GPAT1 | 1.009 | -0.348 | 0.505 | 1.538 | 1.665 | 1.285 | 0.019 | 1.026 | -0.521 | 0.017 | 0.211 | 0.165 | -0.614 | -1.170 | 0.398 | 0.913 |
| SL | PSAP | -1.020 | 0.664 | -0.935 | 1.323 | 0.437 | -0.622 | -0.787 | -0.017 | 0.014 | 0.061 | -0.375 | 0.865 | 1.173 | -0.133 | 0.328 | 0.170 |
|  | GALC | -1.312 | -0.054 | 0.211 | -1.531 | 1.208 | -0.730 | -1.242 | 1.772 | 1.659 | -0.087 | 0.494 | -0.216 | 0.869 | 0.802 | -1.766 | 0.997 |
|  | ARSA | -0.536 | 0.976 | -0.578 | -0.919 | -0.582 | -0.280 | -1.404 | -0.322 | 1.681 | 0.421 | -0.039 | 0.914 | 1.223 | 0.137 | -0.273 | -0.056 |
|  | CGT | -0.611 | -0.197 | 0.167 | -1.865 | 1.017 | 0.693 | 0.511 | 1.312 | 0.221 | 0.182 | -1.050 | -0.086 | 0.060 | 0.809 | -1.711 | -1.847 |
| CER | SPHK2 | 0.645 | 1.101 | 0.345 | -1.478 | -1.490 | 0.551 | 0.625 | -0.479 | 0.105 | -0.688 | 0.170 | -1.447 | -0.697 | 0.516 | -0.209 | -1.711 |
|  | SPHK1 | 0.129 | 0.093 | -1.821 | 1.571 | -1.772 | 1.554 | 0.664 | 1.395 | -0.058 | -0.728 | 2.211 | 1.674 | 0.408 | 0.148 | -0.643 | -0.630 |
|  | ASAH2 | -1.278 | -1.132 | 0.071 | 2.022 | 0.956 | 0.125 | -0.560 | 1.063 | 0.219 | -0.456 | -0.257 | -0.080 | -0.033 | -0.769 | -0.501 | -0.911 |
|  | ASAH1 | -0.931 | -0.057 | -1.012 | 0.737 | 0.354 | -0.061 | -0.598 | 0.519 | -0.009 | 0.966 | -0.755 | 0.614 | 1.726 | 0.570 | 0.264 | 0.914 |
|  | ASM | -0.577 | 0.408 | -0.792 | 1.018 | 0.481 | -0.456 | -1.091 | 0.116 | 0.563 | 0.471 | -0.301 | 0.780 | 0.836 | 0.050 | -0.363 | -0.366 |
|  | CERS2 | -0.752 | -0.872 | 0.000 | -0.291 | 0.312 | 0.490 | -0.386 | 0.397 | -0.429 | 1.893 | 0.278 | 1.955 | 0.230 | -0.974 | -0.523 | -0.671 |
|  | CERS1 | -0.189 | 1.274 | 0.017 | -2.520 | -0.450 | -1.596 | 0.047 | -0.433 | 1.760 | -0.067 | -0.263 | -0.557 | 2.184 | 0.930 | -0.174 | -1.049 |

**Supplementary Table 6. Mean values and standard errors corresponding to the heatmap in Fig.5**

|  |  | 6m | | | | 12m | | | |
| --- | --- | --- | --- | --- | --- | --- | --- | --- | --- |
|  |  | ApoE3-CON | ApoE3-DHA | ApoE4-CON | ApoE4-DHA | ApoE3-CON | ApoE3-DHA | ApoE4-CON | ApoE4-DHA |
| TAG | MGLL | -0.5500±0.5336 | 0.3483±0.4270 | 0.6723±0.6175 | 0.4128±0.2149 | -1.020±0.5609 | -0.5878±0.5144 | 0.1760±0.3728 | 0.5488±0.3646 |
|  | HSL | -0.1423±0.5703 | -0.6380±0.4556 | -0.7833±0.4622 | -0.6030±0.4410 | 0.7253±0.4121 | 1.065±0.4094 | 0.5193±0.2706 | -0.1438±0.4429 |
|  | DGAT1 | -0.1280±0.3253 | 0.8428±0.3882 | -0.0925±0.5834 | -0.3005±0.6281 | -0.2850±0.3573 | -0.6793±0.5002 | -0.0550±0.6427 | 0.6963±0.5153 |
|  | GPAT1 | -0.5708±0.8114 | 0.1665±0.3640 | -1.108±0.5453 | -0.0125±0.1589 | 0.6760±0.4012 | 0.9988±0.3520 | -0.0320±0.1682 | -0.1183±0.4728 |
| SL | PSAP | 0.3090±0.3654 | -0.8565±0.2891 | 0.6008±0.2870 | -0.3400±1.135 | 0.0080±0.5849 | -0.2473±0.2818 | 0.1413±0.2603 | 0.3845±0.2797 |
|  | GALC | 0.2520±0.4043 | 0.4090±0.2247 | -0.3763±0.5263 | -0.5533±0.5807 | -0.6715±0.4387 | 0.2520±0.7315 | 0.4625±0.4277 | 0.2255±0.6651 |
|  | ARSA | 0.5188±0.5479 | -0.9515±0.2014 | 0.9808±0.5144 | -0.6390±0.5926 | -0.2643±0.4222 | -0.6470±0.2610 | 0.7443±0.3679 | 0.2578±0.3325 |
|  | CGT | 0.1110±0.4266 | 0.8768±0.4720 | -0.2700±0.3827 | -0.1190±0.7247 | -0.6265±0.4424 | 0.8833±0.1771 | -0.1833±0.2969 | -0.6723±0.6576 |
| CER | SPHK2 | -0.2278±0.3046 | 0.8525±0.3075 | 0.2833±0.3938 | 0.1270±0.9568 | 0.1533±0.5655 | -0.1983±0.4989 | -0.4650±0.3810 | -0.5253±0.4672 |
|  | SPHK1 | 0.5348±0.3359 | -0.4273±0.3900 | -0.5618±0.1913 | -0.5943±0.2673 | -0.0070±0.6958 | 0.4603±0.7689 | 0.7748±0.6966 | -0.1793±0.2693 |
|  | ASAH2 | 0.4050±0.5035 | 0.3068±0.4928 | -0.8085±0.7239 | 0.4765±0.5639 | -0.0793±0.7628 | 0.3960±0.3814 | -0.1435±0.1432 | -0.5535±0.1932 |
|  | ASAH1 | 0.2905±0.7005 | -0.8353±0.1755 | 0.0833±0.2362 | -0.3493±1.006 | -0.3158±0.4122 | 0.0535±0.2491 | 0.2040±0.3779 | 0.8685±0.3152 |
|  | ASM | 0.6085±0.5374 | -0.9225±0.2177 | 0.5970±0.2877 | -0.4765±1.070 | 0.0143±0.4245 | -0.2375±0.3437 | 0.3783±0.2355 | 0.0393±0.2830 |
|  | CERS2 | 0.2625±0.4923 | -0.8400±0.6374 | 0.2948±0.2548 | 0.1180±0.8409 | -0.4788±0.2028 | 0.2033±0.1998 | 0.9243±0.5951 | -0.4845±0.2560 |
|  | CERS1 | 0.8170±0.4962 | -0.0528±0.3398 | 0.0738±0.3225 | -0.5668±0.3190 | -0.3545±0.7909 | -0.6080±0.3489 | 0.2183±0.5237 | 0.4728±0.6995 |

**Supplementary Table 7. Common differential lipid raft proteins across the four mouse groups after 6 or 12 months of DHA dietary intervention**

|  | UniProt ID | Protein name | Gene name | Change | Subcellular localization |
| --- | --- | --- | --- | --- | --- |
| 1 | Q8VCE6 | 5'(3')-deoxyribonucleotidase, mitochondrial | *Nt5m* | Up | Inner mitochondrial membrane |
| 2 | Q62048 | Astrocytic phosphoprotein PEA-15 | *Pea15* | Up | Inner mitochondrial membrane |
| 3 | P12658 | Calbindin | *Calb1* | Up | Inner mitochondrial membrane |
| 4 | Q08331 | Calretinin | *Calb2* | Up | Inner mitochondrial membrane |
| 5 | Q8BGU2 | Cerebellin-2 | *Cbln2* | Down | Inner mitochondrial membrane |
| 6 | P01756 | Ig heavy chain V region MOPC 104E | *-* | Down | Inner mitochondrial membrane |
| 7 | P01872 | Immunoglobulin heavy constant mu | *Ighm* | Down | Inner mitochondrial membrane |
| 8 | Q9JIA1 | Leucine-rich glioma-inactivated protein 1 | *Lgi1* | Down | Inner mitochondrial membrane |
| 9 | Q99PH1 | Leucine-rich repeat-containing protein 4 | *Lrrc4* | Down | Cell membrane |

**Supplementary Table 8. Sample sizes for each experiment**

| Items | Sample size | Notes |
| --- | --- | --- |
| ApoE3-CON | *N* = 18 (6-month: *N* = 7; 12-month: *N* = 11) | Number of animals per group. Sample sizes for Morris Water Maze test, body weight monitoring and food intake analysis. |
| ApoE3-DHA | *N* = 19 (6-month: *N* = 7; 12-month: *N* = 12) |  |
| ApoE4-CON | *N* = 18 (6-month: *N* = 7; 12-month: *N* = 11) |  |
| ApoE4-DHA | *N* = 19 (6-month: *N* = 9; 12-month: *N* = 10) |  |
| ApoE levels | *N* = 4 | Number of replicate measurements per group. |
| Aβ_1-42_, Aβ_1-40_ and Aβ_1-40_/Aβ_1-42_ ratios | *N* = 4 | Number of replicate measurements per group. |
| Western blot | *N* ≥ 3 | At least three replicate experiments were performed for each indicator. |
| Nissl staining | *N* = 9 | Three animals from each group were selected for staining. Three regions of interest (ROIs) were chosen in the corresponding brain region of each animal. Consequently, 9 ROIs per brain region per group for each indicator were included in the analysis. |
| GFAP expression | *N* = 9 |  |
| Iba1 expression | *N* = 9 |  |
| Lipidomic | *N* = 4 | Four animals were included in each group, and the collected samples were subjected to lipidomic detection and data analysis. |
| Quantitative Real-Time PCR | *N* = 6 | Six biological replicates per group were tested for data analysis. |
| Proteomic | *N* = 4 | Four animals were included in each group, and the collected samples were subjected to proteomic detection and data analysis. |
| Transmission electron microscopy | *N* = 3 | Brain tissues from three animals per group were subjected to electron microscopy detection and subsequent data analysis. |

**Supplementary Table 9. Ingredients of intervention feed and energy contribution ratio (%).**

| **Ingredients** | **Control diet** | | **DHA fortified diet** | |
| --- | --- | --- | --- | --- |
|  | **g/kg** | **kcal/kg** | **g/kg** | **kcal/kg** |
| Casein | 220 | 880 | 220 | 880 |
| L-Cystine | 3.3 | 13.2 | 3.3 | 13.2 |
| Corn Starch | 360.192 | 1441 | 360.192 | 1441 |
| Maltodextrin | 125 | 500 | 125 | 500 |
| Sucrose | 100 | 400 | 100 | 400 |
| Cellulose | 50 | 0 | 50 | 0 |
| Tertiary butylhydroquinone | 0.0054 | 0 | 0.0054 | 0 |
| Complex Minerals | 35 | 0 | 35 | 0 |
| Hydrocholine bitartrate | 2.5 | 0 | 2.5 | 0 |
| Complex Vitamin | 10 | 40 | 10 | 40 |
| Lard | 37 | 333 | 37 | 333 |
| Corn Oil | 27 | 243 | 7 | 63 |
| Fish oil | 0 | 0 | 33.3 | 180 |
| **Total** | 969.9974 | 3850.2 | 983.2974 | 3850.2 |
| **Energy ratio** | **gm (%)** | **kcal (%)** | **gm (%)** | **kcal (%)** |
| Protein | 23 | 23.2 | 23 | 23.2 |
| Carbohydrate | 61.4 | 61.8 | 60.5 | 61.8 |
| Fat | 6.6 | 15.0 | 6.5 | 15.0 |
| **Total** |  | 100 |  | 100 |

**Supplementary Table 10. Primary antibodies used for western blotting**

| Antibody | Brand | Catalogue Number | Dilution |
| --- | --- | --- | --- |
| LRP1 | Abcam | ab92544 | 1: 50000 |
| BACE1 | Abcam | ab183612 | 1: 1000 |
| APP | Proteintech | 60342-1-Ig | 1: 1000 |
| IDE | Abcam | ab133561 | 1: 5000 |
| tau | Proteintech | 66499-1-Ig | 1: 1000 |
| p-tau | Abcam | ab32057 | 1: 1000 |
| GAPDH | Zsbio | TA-08 | 1: 2000 |
| β-actin | Servicebio | GB15003 | 1: 1000 |
| Anti-rabbit IgG (H+L) | ImmunoWay | RS0001 | 1: 20000 |
| Anti-mouse IgG (H+L) | ImmunoWay | RS0002 | 1: 20000 |

LRP1, low-density lipoprotein receptor-related protein 1; BACE1, β-site APP cleaving enzyme 1, also known as β-secretase; APP, amyloid precursor protein; IDE, insulin-degrading enzyme; p-tau, phosphorylated tau; GAPDH, glyceraldehyde-3-phosphate dehydrogenase.

**Supplementary Table 11. Quantification data of Western blot bands**

|  |  | duplicate1 | duplicate2 | duplicate3 | duplicate4 | duplicate5 | duplicate6 |
| --- | --- | --- | --- | --- | --- | --- | --- |
| LRP1 | 6m-E3-CON | 1.2862 | 1.1956 | 0.7719 | 0.5844 | 1.2851 | 0.8767 |
|  | 6m-E3-DHA | 0.8164 | 1.1010 | 0.6581 | 0.6585 | 1.0462 | 0.5689 |
|  | 6m-E4-CON | 0.7534 | 0.9462 | 0.5811 | 0.5134 | 0.8377 | 0.5805 |
|  | 6m-E4-DHA | 0.7290 | 0.8431 | 0.5317 | 0.5405 | 0.6740 | 0.4875 |
|  | 12m-E3-CON | 0.7402 | 0.8934 | 0.5141 | 0.4594 | 0.7097 | 1.0994 |
|  | 12m-E3-DHA | 0.6693 | 0.4987 | 0.3970 | 0.4671 | 0.9098 | 0.9615 |
|  | 12m-E4-CON | 0.7491 | 0.9324 | 0.7551 | 0.3774 | 1.0553 | 1.2945 |
|  | 12m-E4-DHA | 1.2772 | 0.7067 | 0.8028 | 0.5037 | 0.7824 | 0.6842 |
| BACE1 | 6m-E3-CON | 0.7150 | 0.7051 | 0.8768 | 1.7032 |  |  |
|  | 6m-E3-DHA | 1.5350 | 0.9612 | 1.3925 | 2.2612 |  |  |
|  | 6m-E4-CON | 1.4323 | 1.1582 | 1.5732 | 2.6763 |  |  |
|  | 6m-E4-DHA | 1.4972 | 1.0954 | 1.0623 | 2.3417 |  |  |
|  | 12m-E3-CON | 1.3288 | 1.3091 | 1.4539 | 1.6706 |  |  |
|  | 12m-E3-DHA | 1.1291 | 1.0361 | 1.0208 | 1.6882 |  |  |
|  | 12m-E4-CON | 1.2518 | 0.8230 | 1.6342 | 1.7526 |  |  |
|  | 12m-E4-DHA | 0.7772 | 0.7692 | 0.9754 | 1.5713 |  |  |
| APP | 6m-E3-CON | 0.9411 | 1.3834 | 0.6395 | 1.0360 |  |  |
|  | 6m-E3-DHA | 0.3174 | 0.3337 | 0.1820 | 0.3797 |  |  |
|  | 6m-E4-CON | 0.4976 | 0.3990 | 0.2328 | 0.4299 |  |  |
|  | 6m-E4-DHA | 0.6038 | 0.5198 | 0.2412 | 0.5534 |  |  |
|  | 12m-E3-CON | 1.3105 | 0.9525 | 0.4395 | 1.1016 |  |  |
|  | 12m-E3-DHA | 0.8930 | 0.3692 | 0.2693 | 0.5585 |  |  |
|  | 12m-E4-CON | 0.8206 | 0.4094 | 0.1669 | 0.4623 |  |  |
|  | 12m-E4-DHA | 0.6300 | 0.2945 | 0.1756 | 0.5268 |  |  |
| IDE | 6m-E3-CON | 0.8836 | 0.6645 | 1.1300 | 1.3219 |  |  |
|  | 6m-E3-DHA | 0.8057 | 0.6432 | 1.3160 | 1.4138 |  |  |
|  | 6m-E4-CON | 0.7974 | 1.1673 | 1.1763 | 1.4332 |  |  |
|  | 6m-E4-DHA | 0.9818 | 1.1628 | 1.0239 | 1.1573 |  |  |
|  | 12m-E3-CON | 1.2113 | 1.5019 | 0.9965 | 1.1076 |  |  |
|  | 12m-E3-DHA | 1.2328 | 1.6011 | 1.3020 | 1.3456 |  |  |
|  | 12m-E4-CON | 1.2224 | 1.9889 | 1.5943 | 1.2441 |  |  |
|  | 12m-E4-DHA | 0.9079 | 1.1019 | 0.8507 | 1.0087 |  |  |
| p-tau | 6m-E3-CON | 1.0062 | 1.1877 | 1.0166 |  |  |  |
|  | 6m-E3-DHA | 2.8785 | 3.2342 | 3.5791 |  |  |  |
|  | 6m-E4-CON | 2.0804 | 2.1573 | 1.6305 |  |  |  |
|  | 6m-E4-DHA | 1.9018 | 1.8321 | 1.5689 |  |  |  |
|  | 12m-E3-CON | 1.8236 | 3.1263 | 1.8691 |  |  |  |
|  | 12m-E3-DHA | 1.8872 | 2.3296 | 1.8267 |  |  |  |
|  | 12m-E4-CON | 1.7986 | 2.2091 | 2.0801 |  |  |  |
|  | 12m-E4-DHA | 1.4991 | 1.2190 | 1.8736 |  |  |  |
| tau | 6m-E3-CON | 1.0445 | 0.9613 | 0.9941 |  |  |  |
|  | 6m-E3-DHA | 0.8252 | 0.8954 | 1.0056 |  |  |  |
|  | 6m-E4-CON | 0.9997 | 0.9666 | 0.9018 |  |  |  |
|  | 6m-E4-DHA | 0.9134 | 0.8476 | 0.8646 |  |  |  |
|  | 12m-E3-CON | 1.0014 | 0.9468 | 0.8807 |  |  |  |
|  | 12m-E3-DHA | 1.0155 | 0.8194 | 0.8921 |  |  |  |
|  | 12m-E4-CON | 0.8722 | 0.8841 | 0.9264 |  |  |  |
|  | 12m-E4-DHA | 0.9243 | 0.8810 | 0.9576 |  |  |  |
| p-tau/tau | 6m-E3-CON | 0.9633 | 1.2354 | 1.0226 |  |  |  |
|  | 6m-E3-DHA | 3.4883 | 3.6119 | 3.5591 |  |  |  |
|  | 6m-E4-CON | 2.0811 | 2.2319 | 1.8082 |  |  |  |
|  | 6m-E4-DHA | 2.0821 | 2.1616 | 1.8145 |  |  |  |
|  | 12m-E3-CON | 1.8210 | 3.3020 | 2.1222 |  |  |  |
|  | 12m-E3-DHA | 1.8585 | 2.8432 | 2.0477 |  |  |  |
|  | 12m-E4-CON | 2.0621 | 2.4988 | 2.2454 |  |  |  |
|  | 12m-E4-DHA | 1.6218 | 1.3836 | 1.9566 |  |  |  |

**Supplementary Table 12. The primers used for RT-qPCR**

| Primers | Forward/Reverse | Sequence (5’ - 3’) |
| --- | --- | --- |
| *Dgat1* | Dgat1-F | TCTGCTACGACGAGTTCTTGAGATG |
|  | Dgat1-R | AAGGGCTTCATGGAGTTCTGGATAG |
| *Atgl* | Atgl-F | CATCCAGTTCAACCTTCGCAATCTC |
|  | Atgl-R | AAGTCCATCTCTGTAGCCCTGTTTG |
| *nSMase2* | nSMase2-F | CGTGCGGCTGCTAAGTTGAAAG |
|  | nSMase2-R | CGGCTGGCGAAGAAGAGACC |
| *Cers3* | Cers3-F | GCCCTTCTTCTCCTACATCTTCCTC |
|  | Cers3-R | TTCCTCTTCGTTGTCACTCCTCAC |
| *Acer3* | Acer3-F | AGACTGGAGAAGCGGTACATTGC |
|  | Acer3-R | AGAGTCATGTGGAAACACCAGGATC |
| *Sgms2* | Sgms2-F | TGTGCGGAGACTTCCTCTTCAG |
|  | Sgms2-R | TCAGCAGCCAGCAGACCAAG |
| *Gal3st1* | Gal3st1-F | GCATCAAGTCCATCCTGGGTTAC |
|  | Gal3st1-R | AGATTGGCACCGAGGTCAGAC |
| *Arsa* | Arsa -F | CCACCACACTCACTACCCTCAG |
|  | Arsa-R | CGCATCAACTCAGGACCGTTATC |
| *Psap* | Psap -F | GTACGCCCTCGCCCTCTTC |
|  | Psap-R | TCCACCGCCGTCTTCACATC |
| *Lpin1* | Lpin1-F | AGCCAACCAGTCACCACAGTC |
|  | Lpin1-R | GCCACCGCAGAGGGAGATG |

nSMase2: neutral Sphingomyelinase 2; Cers3: Ceramide Synthase 3; Acer3: Alkaline Ceramidase 3; Sgms2: Sphingomyelin Synthase 2; Lpin1: Lipin 1; Dgat1: Diacylglycerol Acyltransferase 1; Atgl: Adipose Triglyceride Lipase; Gal3st1: Galactose-3-O-Sulfotransferase 1; Arsa: Arylsulfatase A; Psap: Prosaposin.

**Supplementary Table 13. List of internal standards used for lipidomic**

| d_9_-PC32:0 (16:0/16:0) | d_9_-PC36:1p (18:0p/18:1) | d_7_-PE33:1 (15:0/18:1) | d_9_-PE36:1p (18:0p/18:1) | d_31_-PS (d31-16:0/18:1) | d_7_-PA33:1 (15:0/18:1) |
| --- | --- | --- | --- | --- | --- |
| d_7_-PG33:1 (15:0/18:1) | d_7_-PI33:1 (15:0/18:1) | C17-SL | d_5_-CL72:8 (18:2)4 | Cer d18:1/15:0-d_7_ | d_9_-SM d18:1/18:1 |
| C8-GluCer | C8-GalCer | d_3_-LacCer d18:1/16:0 | Gb3 d18:1/17:0 | d_7_-LPC18:1 | d_7_-LPE18:1 |
| C17-LPI | C17-LPA | C17-LPS | C17-LPG | d17:1 Sph | d17:1 S1P |
| C14-BMP | d_3_-16:0-carnitine | GM3-d18:1/18:0-d3 | d_31_-16:0 | d_8_-20:4 | TAG(14:0)3-d_5_ |
| TAG(16:0)3-d_5_ | TAG(18:0)3-d_5_ | d_5_-DAG17:0/17:0 | d_5_-DAG18:1/18:1 | d_6_-cholesterol | d_6_-C18:0 cholesteryl ester |

**Supplementary Figure 1 Original Western blot bands**
